# Study on the Effect of Macrophages on Vascular Endothelium in Mice With Different TCM Syndromes of Dyslipidemia and its Biological Basis Based on RNA-Seq Technology

**DOI:** 10.3389/fphar.2021.665635

**Published:** 2021-08-26

**Authors:** Jing Chen, Chao Ye, Zheng Yang, Tieshan Wang, Bing Xu, Pengyang Li, Shan Zhang, Xiaolin Xue

**Affiliations:** ^1^ Preventive Treatment of Disease Department, The Third Affiliated Hospital, Beijing University of Chinese Medicine, Beijing, China; ^2^ Orthopedics Department, Dongzhimen Hospital, Beijing University of Chinese Medicine, Beijing, China; ^3^ School of Traditional Chinese Medicine, Beijing University of Chinese Medicine, Beijing, China; ^4^ Beijing Research Institute of Chinese Medicine, Beijing University of Chinese Medicine, Beijing, China; ^5^ Traditional Chinese Medicine Department, Tibetology Research Center of Beijing Tibetan Medicine Hospital, Beijing, China

**Keywords:** macrophages, traditonal Chinese medicine, dyslipidemia, phlegm-dampness retention syndrome, spleen and kidney Yang deficiency syndrome, RNA-Seq

## Abstract

**Background:** “Treating the same disease with different methods” is a Traditional Chinese medicine (TCM) therapeutic concept suggesting that, while patients may be diagnosed with the same disease, they may also have different syndromes that require distinct drug administrations.

**Objective:** This study aimed to identify the differentially expressed genes and related biological processes in dyslipidemia in relation to phlegm–dampness retention (PDR) syndrome and spleen and kidney Yang deficiency (SKYD) syndrome using transcriptomic analysis.

**Methods:** Ten ApoE^−/−^ mice were used for the establishment of dyslipidemic disease–syndrome models via multifactor-hybrid modeling, with five in the PDR group and five in the SKYD group. Additionally, five C57BL/6J mice were employed as a normal control group. Test model-quality aortic endothelial macrophages in mice were screened using flow cytometry. Transcriptomic analysis was performed for macrophages using RNA-Seq.

**Results:** A quality assessment of the disease–syndrome model showed that levels of lipids significantly increased in the PDR and SKYD groups, compared to the normal control group, *p* < 0.05. Applying, in addition, hematoxylin and eosin staining of aorta, the disease model was also successfully established. A quality assessment of the syndrome models showed that mice in the PDR group presented with typical manifestations of PDR syndrome, and mice in the SKYD group had related manifestations of SKYD syndrome, indicating that the syndrome models were successfully constructed as well. After comparing the differentially expressed gene expressions in macrophages of the dyslipidemic mice with different syndromes, 4,142 genes were identified with statistical significance, *p* < 0.05. Gene ontology analysis for the differentially expressed genes showed that the biological process of difference between the PDR group and the SKYD group included both adverse and protective processes.

**Conclusion:** The differentially expressed genes between PDR syndrome and SKYD syndrome indicate different biological mechanisms between the onsets of the two syndromes. They have distinctive biological processes, including adverse and protective processes that correspond to the invasion of pathogenic factors into the body and the fight of healthy Qi against pathogenic factors, respectively, according to TCM theory. Our results provide biological evidence for the TCM principle of “treating the same disease with different treatments.”

## Introduction

As life quality increases and dietary patterns change, multiple factors are beginning to give rise to a growing morbidity of dyslipidemia in humans. A recent epidemiological study has shown that dyslipidemia is closely linked to cardiovascular and cerebrovascular diseases, including coronary atherosclerotic heart disease and cerebral infarction (Li et al., 2019). It is estimated that the overall morbidity of dyslipidemia in Chinese adults has reached 40.40% and is continuing to rise (Chu et al., 2016).

**GRAPHICAL ABSTRACT:**
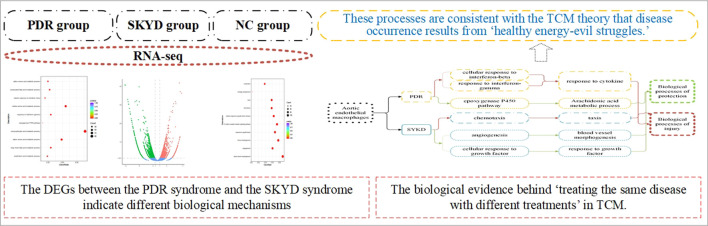


Dyslipidemia is characterized by abnormalities in the quantity and quality of lipids in the plasma, including lower high-density lipoprotein cholesterol (HDL-C) levels and higher triglyceride (TG), total cholesterol (TC), and low-density lipoprotein cholesterol (LDL-C) levels (Sarzynski et al., 2015). Lipid and cholesterol accumulation (Pantos et al., 2007) in the vascular wall may lead to endothelial dysfunction. Dyslipidemia is an independent and changeable risk factor shortening the onset time of atherosclerosis (Ciccone et al., 2011). The 2013 American College Foundation of Cardiology and American Heart Association guidelines on the management of blood lipids in atherosclerotic cardiovascular disease recommended that management of dyslipidemia is the key to controlling risk factors for ischemic cardiovascular events (Stone et al., 2014).

Numerous clinical studies and laboratory experiments have ascertained the satisfactory efficacy of TCM with respect to dyslipidemia, enriching the therapies available for the disease (He et al., 2018). Taking berberine orally has been shown to lower TG and cholesterol levels (Hu et al., 2012), and the oral administration of *Monascus* rice has also been found to significantly reduce the levels of blood lipids and to be well tolerated (Lin et al., 2005). An important effect of TCM lies in the accurate discrimination of TCM syndromes of dyslipidemia, which is the basis of TCM diagnosis and treatment. The differentiation of syndromes is brought about through the collection of clinical information (i.e., “TCM symptoms”) of patients via integrated TCM diagnostic methods, including looking, listening, and questioning the individual, and feeling their pulse, as well as analyzing and summarizing the etiological factors and pathogenesis according to the TCM theoretical approach.

As individuals present with different TCM symptoms, dyslipidemic patients may exhibit various syndromes that can be considered different TCM subtypes of dyslipidemia. For example, spleen and kidney Yang deficiency (SKYD) syndrome and phlegm–dampness retention (PDR) syndrome are both common in patients with dyslipidemia. In addition, the vascular endothelium is responsible for delivering nutrients in a dynamic way (Eelen et al., 2020), while dyslipidemia acts as a risk factor of cardiovascular disease, potentially by promoting endothelium dysfunction—a prerequisite for the occurrence of atherosclerotic manifestations (Scicchitano et al., 2019). A critical mechanism of endothelium dysfunction is oxidative stress. Excessive nitric oxide (NO) binding with hyperoxides can form a peroxynitrite anion (ONOO−), and ONOO− triggers oxidative stress of the vascular endothelium and endothelial injury through its oxidative effect (nitrification) on proteins, exacerbating the endothelial injury. Accordingly, the severity of endothelial injury can be indicated by ONOO− levels.

Our team has been studying the integration of TCM differentiation and Western medicine diagnosis alongside investigating endothelial injury differences as the characteristics between SKYD syndrome and PDR syndrome in dyslipidemia. Findings from our previous research have suggested that dyslipidemic patients with SKYD syndrome and PDR syndrome exhibit different serum ONOO− concentrations, reflecting the difference in TCM syndromes in a certain sense (Chen et al., 2016). Consequently, we theorize that different syndromes of dyslipidemia may correspond to different degrees of endothelial injury. In dyslipidemia, macrophages play a pivotal role in the process of endothelial injury. Recognizing this critical effect of macrophages, and following on from our previous study series, the present study focuses on aortic endothelial macrophages and explores the characteristics of the endothelial injury between individuals with SKYD syndrome and those with PDR syndrome as well as dyslipidemia.

In dyslipidemia, monocytes that migrate to the endothelium differentiate into macrophages via macrophage colony stimulating factors, and then damage the vascular endothelium. Macrophages consist of two subtypes, M1 and M2 macrophages (Davies and Taylor, 2015). Lactate dehydrogenase stimulates the expression of adhesion molecules and chemokines, thereby differentiating monocytes into macrophages through various pathways. M1 macrophages can release inflammatory factors that facilitate the progression of inflammation and further impair vascular endothelial cells (Pesce et al., 2009). M2 macrophages release anti-inflammatory factors that are involved in angiogenesis and tissue growth and delay the progression of inflammation (Moore and West, 2019). A prior study established that macrophages are indispensable in the formation of atherosclerosis (Yamada et al., 1998). Moreover, according to TCM theory, the two subtypes exhibit antagonistic characteristics, corresponding to “healthy Qi” (the ability to combat evils, preventing disease) versus “evil Qi” (pathogenetic factors, factors damaging healthy Qi). The interactions between healthy Qi and evil Qi are characterized by “healthy energy–evil struggles, mutually opposing and constraining, and the rule of waxing and waning” in the occurrence and development of diseases and syndromes.

Disease–syndrome combination is a significant mode for TCM diagnosis and treatment in the clinic. Furthermore, in this context, the in-depth investigation of diseases and syndromes requires biological studies using disease–syndrome animal models in agreement with the traits of TCM theories to apprehend the underlying mechanisms. The characteristics of such models, such as rigorous controls, high repeatability, and success rates, make it easier to regulate the research cycle, repetitively perform experiments, collect more data, and comprehensively analyze the data. Hence, the establishment of an appropriate disease–syndrome animal model is increasingly attracting research attention.

An important element of TCM syndrome research is the investigation of biological mechanisms and the characteristics of syndromes using animal models. Accordingly, we developed a disease–syndrome animal model in the present study for analysis. The quality of a disease–syndrome animal model can affect the accuracy and credibility of any study based on it. We employed multifactor-hybrid modeling and established dyslipidemic mouse models featuring SKYD syndrome and PDR syndrome, based on the model used in our previous study, so that the quality of the animal models could be ensured as much as possible. The disease model replicated the core physiopathologic process present during the occurrence and development of a disease, and the syndrome models each simulated the core etiology and pathology during the onset of a syndrome.

Transcriptomics is a technique used to study gene transcription and regulation related to TCM syndromes and is conducive to in-depth biological research toward a better understanding of the pathogenesis of TCM syndromes, and so warrants more prevalent application in TCM syndrome research (Jiang and Li, 2020). Gan et al. (2020) studied Yin deficiency–heat syndrome using transcriptomics technology and obtained its potential biomarkers. Currently, though, studies concerning the characteristics of SKYD syndrome and PDR syndrome in relation to dyslipidemia are rarely reported. Our study focuses on the two-way impacts between the severity of endothelial injury and macrophages, and analyzes the differences in biological processes and signaling pathways between different TCM syndromes (subtypes) of dyslipidemia using transcriptomic techniques. Thus, this study provides a reference for in-depth mechanical research of TCM syndromes and studies of the targets of TCM drugs.

## Materials and Methods

**FIGURE 1:**
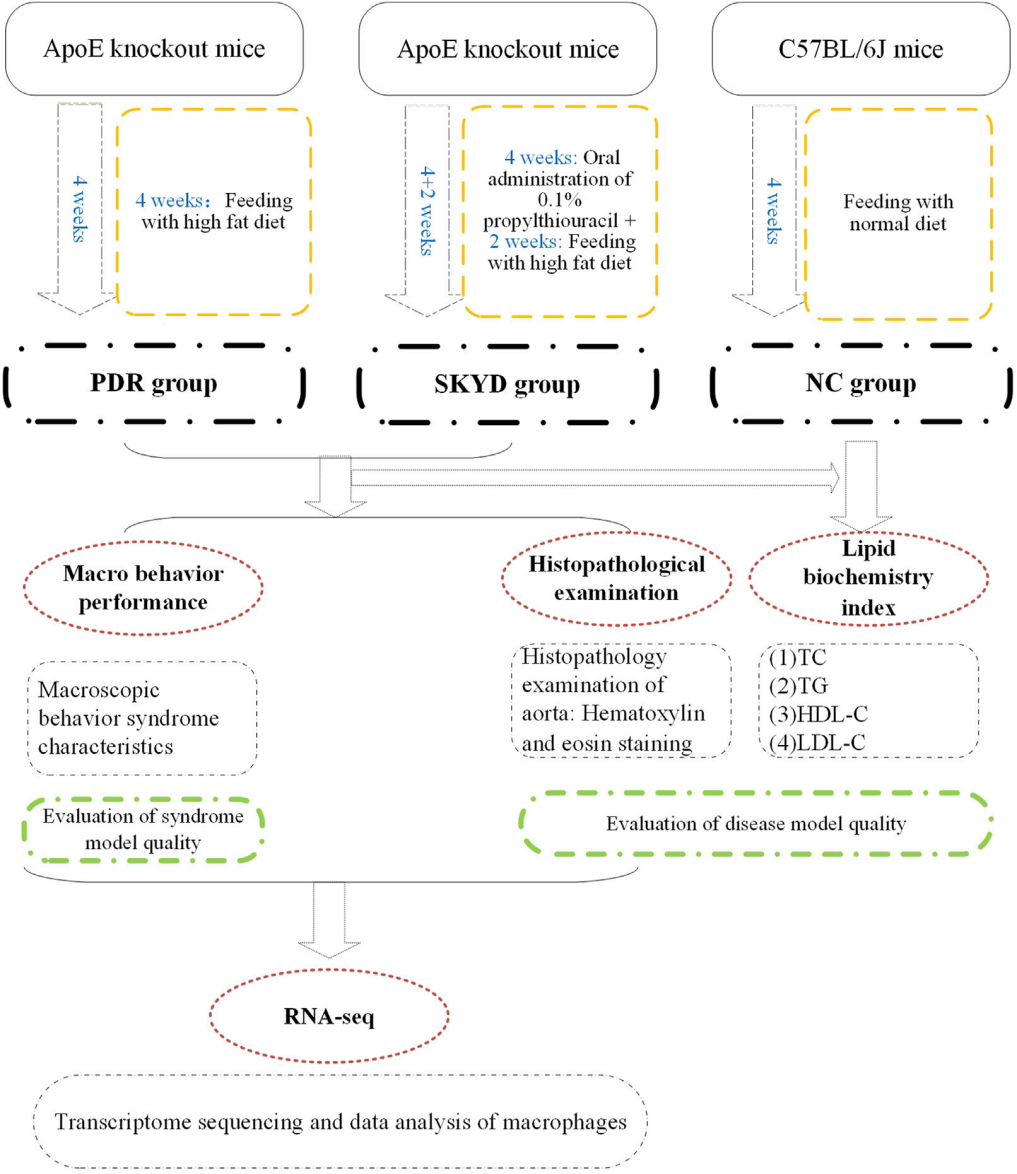
Flowchart of the study.

Figure 1 presents an overview of the study’s materials and methods, described in full below.

### Experimental Animals

The animals featured in the research comprised 10 apolipoprotein E knockout (ApoE^−/−^) mice—male, 6 weeks old, body mass about 20 ± 5 g—and five C57BL/6J mice of the same strain—male, 6 weeks old, body mass about 20 ± 5 g. All animals were raised in the Beijing Changyang Xishan Farm; the rearing environment incorporated a room temperature of 21–25°C, 50%–70% humidity, and 12 h of alternating shade. The ethics of this study were approved by the animal ethics review committee of the Institute of Basic Theories of Chinese medicine, Chinese Academy of Chinese Medical Sciences, approval no. 201908006 (Beijing, China).

### Experimental Reagents and Model-Making Feed

#### Main Reagents for the Experiment

LDL cholesterol test kit (Nanjing Jiancheng Bioengineering Institute Co., Ltd.); total cholesterol lipoprotein test kit (Nanjing Jiancheng Bioengineering Institute Co., Ltd.); HDL cholesterol test kit (Nanjing Jiancheng Bioengineering Institute Co., Ltd.); triglyceride test kit (Nanjing Jiancheng Bioengineering Institute Co., Ltd.); FITC anti-mouse F4/80 antigen (BM8.1) (Tonbo Biosciences Co., Ltd.); PE anti-human/mouse CD11b (M1/70) (Tonbo Biosciences Co., Ltd.); hematoxylin–eosin (HE) staining solution (Aijia Biotechnology Co., Ltd.).

##### Experimental Modeling Feed

The dyslipidemic feed formula consisted of 63.6% basal feed +15% lard +20% sucrose +1.2% cholesterol +0.2% sodium cholate, provided by Beijing Keaoxieli Feed Co.

### Modeling and Evaluation Methods of Animal Models of Diseases and Syndromes

#### Modeling Methods

##### The PDR Group

Five ApoE knockout mice were randomly selected and fed a high-fat diet for 4 weeks, from Weeks 1–4 (the disease model was constructed at 2 weeks, and the syndrome model was constructed at 2 weeks).

##### The SKYD Group

Five ApoE knockout mice were randomly selected and given 0.1% propylthiouracil by gavage at a dose of 10 mg/(kg·d) during Weeks 1–4 (constructing the syndrome model), and high-fat chow for 2 weeks during Weeks 5 and 6 (constructing the disease model).

##### The Normal Control Group

Five C57BL/6J mice were given normal chow for modeling for a total of 4 weeks from Weeks 1–4.

#### Model Evaluation Methods

Model evaluation was completed as follows: 1) evaluation of model quality of dyslipidemia by means of serum lipid index testing and aortic pathology staining; 2) evaluation of model quality of the PDR group and the SKYD group by observing the characteristic manifestations of the syndromes.

### Sampling and Macrophage Screening Methods

Mice were fasted without water for 18 h before sampling and given anesthesia for execution.

#### Animal Serum

Animals in the PDR group, the SKYD group, and the NC group, after taking whole blood, placed in conventional serum tubes, 3,000 rpm for 10 min, separated serum, divided and immediately stored in −80°C refrigerator.

#### Animal Tissues

Aortic tissues were taken from animals in the PDR group and the SKYD group.

#### Macrophages

Aorta of animals from the PDR group and the SKYD group were isolated and removed intact, digested by adding trypsin, sieved and ground, digestion was terminated, centrifuged, supernatant was decanted, re-suspended by adding PBS, centrifuged again, supernatant was decanted, and labeled with FITC anti-mouse F4/80 antigen (BM8.1) and PE anti-human/mouse CD11b (M1/70), blown and re-suspended, and stored away from light. After labeling the macrophages, the macrophages were screened by fluorescence-activated cell sorting (FACS) using the MoFlo XDP Ultra-Fast Flow Cell Sorting System (Beckman Coulter Co., Ltd.). (See Supplementary Material S1).

### Measurement of Indicators

#### General Observation of the Model

Observing and recording mental status, body hair glossiness, movement, feces, etc.

#### Lipid Index Testing

Serum testing of TG, TC lipoprotein, HDL-C, and LDL-C levels in each group.

#### Aortic Histopathology

Specimens were fixed in 10% neutral formalin at room temperature for 24 h. The specimens were fixed for 48 h at room temperature using freshly prepared 4% formaldehyde, followed by paraffin embedding and sectioning. Specimens were cut into 4 mm-thick sections and subjected to histological HE staining to observe the morphological features of the vascular endothelium.

After staining, the slices were photographed and examined using an optical microscope (AE41; Motic) equipped with a digital scanner (Panoramic MIDI; 3DHISTECH) to record images of the stained slices.

#### Macrophage Transcriptomic Sequencing and Data Analysis

Total RNAs of macrophages were extracted in accordance with the TRIzol® manual (Life Technologies, Inc., Gaithersburg, MD, United States). Preparation of library and sequencing of transcriptomes were carried out using Illumina HiSeq X Ten (Novogene Bioinformatics Technology Co., Ltd., Beijing, China). The mapping of 100-bp paired-end reads to genes was undertaken using HTSeq v0.6.0 software, while fragments per kilo base of transcript per million fragments mapped (FPKM) were also analyzed. Raw reads from RNA-Seq libraries were trimmed to remove the adaptor sequence and the reads with adaptor contaminants and low-quality reads (the mass value Q score <5 of the base number accounts for more than 50%) and reads from N (N indicates that the base information that cannot be determined) which was >10%.

After filtering, reference genome and gene model annotation files were downloaded from a genome website browser (NCBI/UCSC/Ensembl). Indexes of the reference genome were built using Bowtie (version 2.0.6) and paired-end clean reads were aligned to the reference genome using TopHat (version 2.0.9). Bowtie was used for a Burrows–Wheeler transform algorithm for mapping reads to the genome, and TopHat can generate a database of splice junctions based on the gene model annotation file and thus achieve a better mapping result than other non-splice mapping tools.

For the quantification of gene expression level, HTSeq (version 0.6.1) was used to count the read numbers mapped for each gene. The reads per kilo base of transcript per million mapped reads (RPKM) of each gene was calculated based on the gene read counts mapped to this gene. A differential expression analysis was performed using the DESeq R package (3.18.1). The *p* values were adjusted using the Benjamini and Hochberg method. A corrected *p* value of 0.05 and absolute fold change of 2 were set as the threshold for significantly differential expression.

Gene ontology (GO) enrichment analysis of differentially expressed genes (DEGs) was implemented using the clusterProfiler R package, in which gene length bias was corrected. GO terms with a corrected *p* value of less than 0.05 were considered significantly enriched by DEGs.

### Statistical Methods

Subject-related data were statistically processed by applying SPSS (version 19.0) statistical software. The measurement data were expressed as the mean plus or minus the standard deviation. A one-way analysis of variance with randomized group design was used for comparison between groups; Fisher’s least significant difference test was used for two-way comparison, if the data variance was the same; Tamhane’s test was used for two-way comparison, if the variance was not the same; and the difference was considered statistically significant at *p* < 0.05. The test level was α = 0.05, and the confidence interval for parameter estimation was 95%.

## Results

### General Information and Macro Behavioral Performance

Ten ApoE knockout mice and five C57BL/6J mice of the same strain without deletion were used in the experiment, with 15 mice entering into the resulting analysis. Before sampling, the mice in the PDR group had exhibited loss of body hair brightness, lethargy, and laziness; the mice in the SKYD group had showed arching of the back and curling up, tranquil, lethargy, lying down, and a propensity to pile up.

### Comparison of Blood Lipid Indexes

Evaluating the lipid indexes, compared with the NC group, TG, TC, and LDL-C levels were found to be significantly higher in the PDR group and the SKYD group, *p* < 0.05. Compared with the NC group, no significant statistical differences were perceived between the HDL-C indexes of the PDR and SKYD groups. No significant statistical differences were seen between the PDR and SKYD groups when comparing TG, TC, and HDL-C levels. Also, no statistically significant differences were found in the comparisons of the TG, TC, HDL-C, LDL-C, and LDL-C indexes between the PDR and SKYD groups. These results are summarized in Tables 1–4.

**TABLE 1:** Total cholesterol index in three groups of mice (mean ± standard deviation).

Group	TG index
PDR group	23.32 ± 2.33^a^
SKYD group	29.32 ± 6.32^b^ ^,^ ^c^
NC group	2.38 ± 0.38
F	65.82
*p*	0.000

a was compared with NC group, *p* <0.05;

b was compared with NC group, *p* <0.05;

c was compared with PDR group, *p >*0.05.

**TABLE 2:** Triglycerides index in three groups of mice (mean ± standard deviation).

Group	TC index
PDR group	2.96 ± 0.11^a^
SKYD group	4.71 ± 1.15^b^ ^,^ ^c^
NC group	0.80 ± 0.79
F	43.07
*p*	0.000

a was compared with NC group, *p* <0.05;

b was compared with NC group, *p* <0.05;

c was compared with PDR group, *p >*0.05;

**TABLE 3:** HDL-C index in three groups of mice (mean ± standard deviation).

Group	HDL-C index
PDR group	5.17 ± 2.15
SKYD group	3.03 ± 1.92^a^
NC group	3.55 ± 0.68
F	2.13
*p*	0.162

Note:

a was compared with PDR group, *p >*0.05;

**TABLE 4:** LDL-C index in three groups of mice (mean ± standard deviation).

Group	LDL-C index
PDR group	2.54 ± 0.22^a^
SKYD group	3.60 ± 1.39^b^ ^,^ ^c^
NC group	0.89 ± 0.12
F	13.88
*p*	0.001

Note:

a was compared with NC group, *p* <0.05;

b was compared with NC group, *p* <0.05

c was compared with PDR group, *p >*0.05.

### Comparison of HE Staining Examination

**FIGURE 2:**
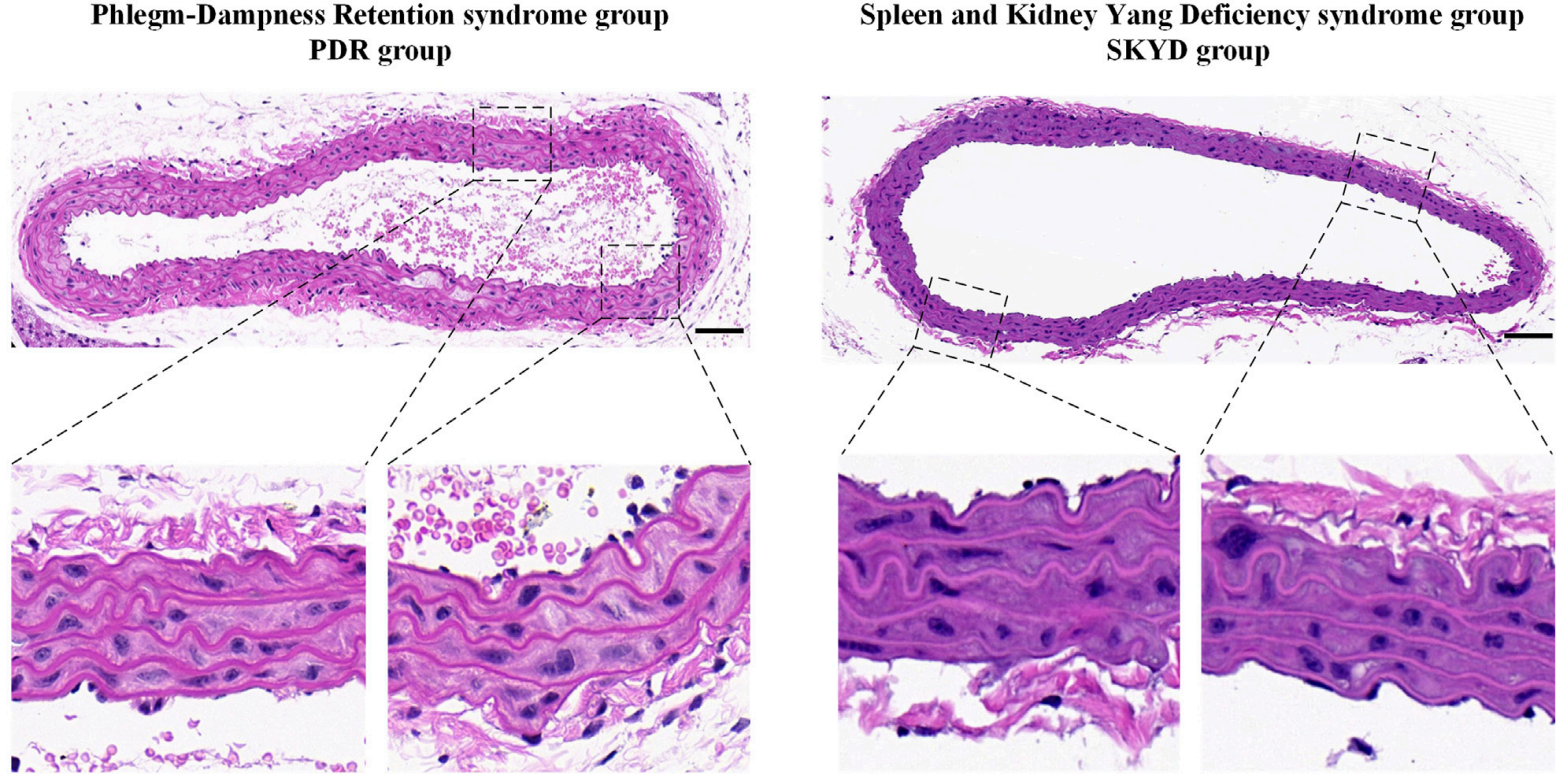
The aorta was stained with hematoxylin and eosin. Scale bars represent 100 μm.

The aortas from the PDR group and the SKYD group were examined using HE staining. The endothelial surface of the vessels was observed to be smooth, the inner, middle, and outer membranes showed clear, and no obvious lipid deposition or lipid streak production was seen. See Figure 2.

### Transcriptome Sequencing Analysis of Macrophages in Mice With Dyslipidemia Syndrome Model

#### Analysis of DEGs in Macrophages of Mice With Different Syndromes of Dyslipidemia Generated by RNA Sequencing

**FIGURE 3:**
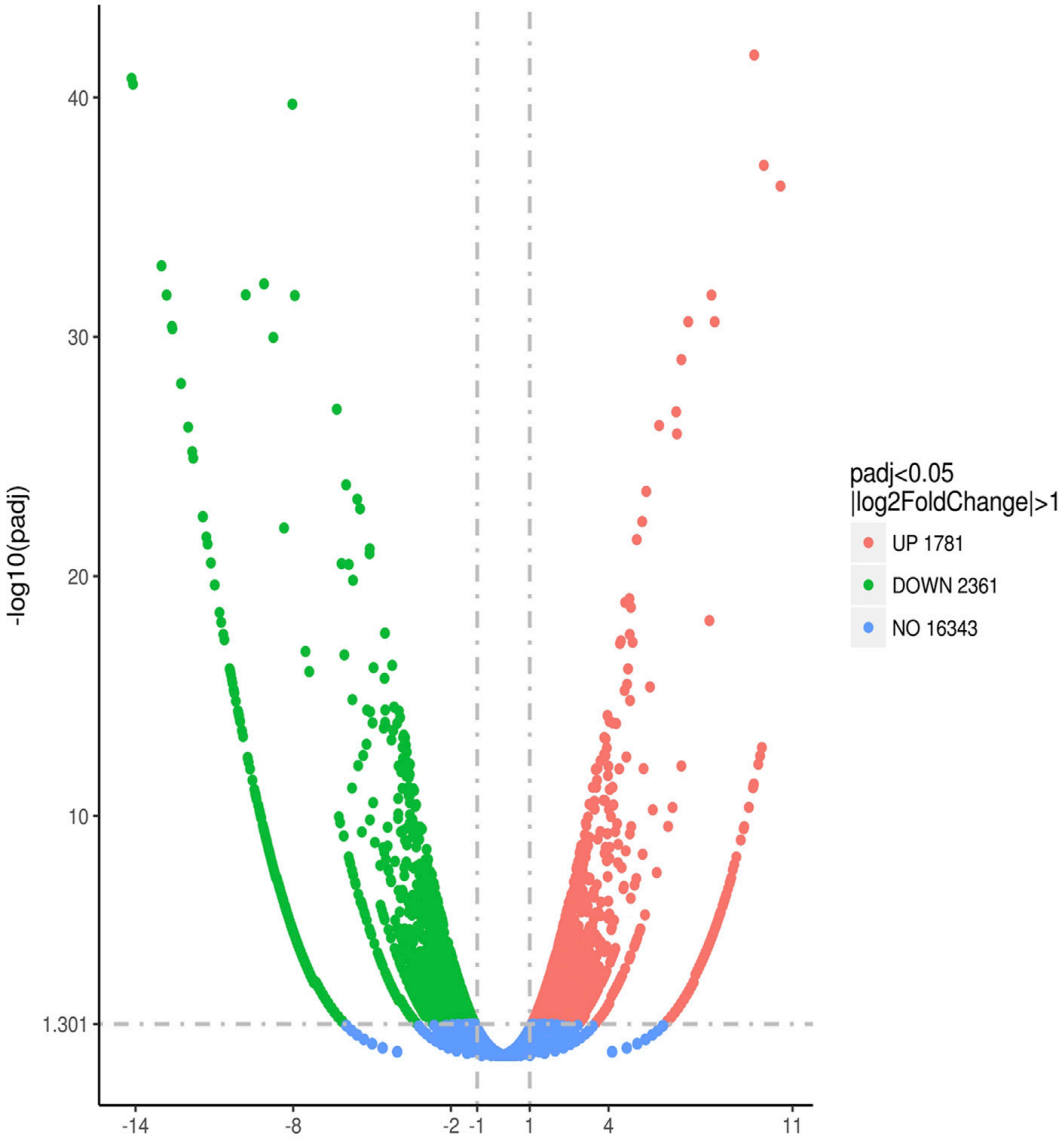
Quantitative comparison of gene expression levels in aortic macrophages of mice with dyslipidemia: PDR group vs. SKYD group.

After comparing the DEG expressions in macrophages in dyslipidemic mice with different syndromes, 4,142 genes were identified with statistical significance, *p* < 0.05. Among them, 1,781 genes were upregulated and 2,361 genes were downregulated. See Figure 3.

Through additional reference to bioinformatics information, a total of 284 DEGs were selected in this study for in-depth analysis of content related to vascular endothelial injury. In a comparison of the 74 DEGs between the PDR group and the SKYD group, the number of reads in the PDR group was statistically higher than that in the SKYD group, *p* < 0.05. See Supplementary Material S2.

#### GO Enrichment Analysis of DEGs Associated With Vascular Endothelial Injury in Macrophages

Through GO enrichment analysis based on DEGs, macrophage sequencing data of mice in the PDR group and the SKYD group were analyzed, focusing on the enrichment results of biological processes related to vascular endothelial injury.

**FIGURE 4:**
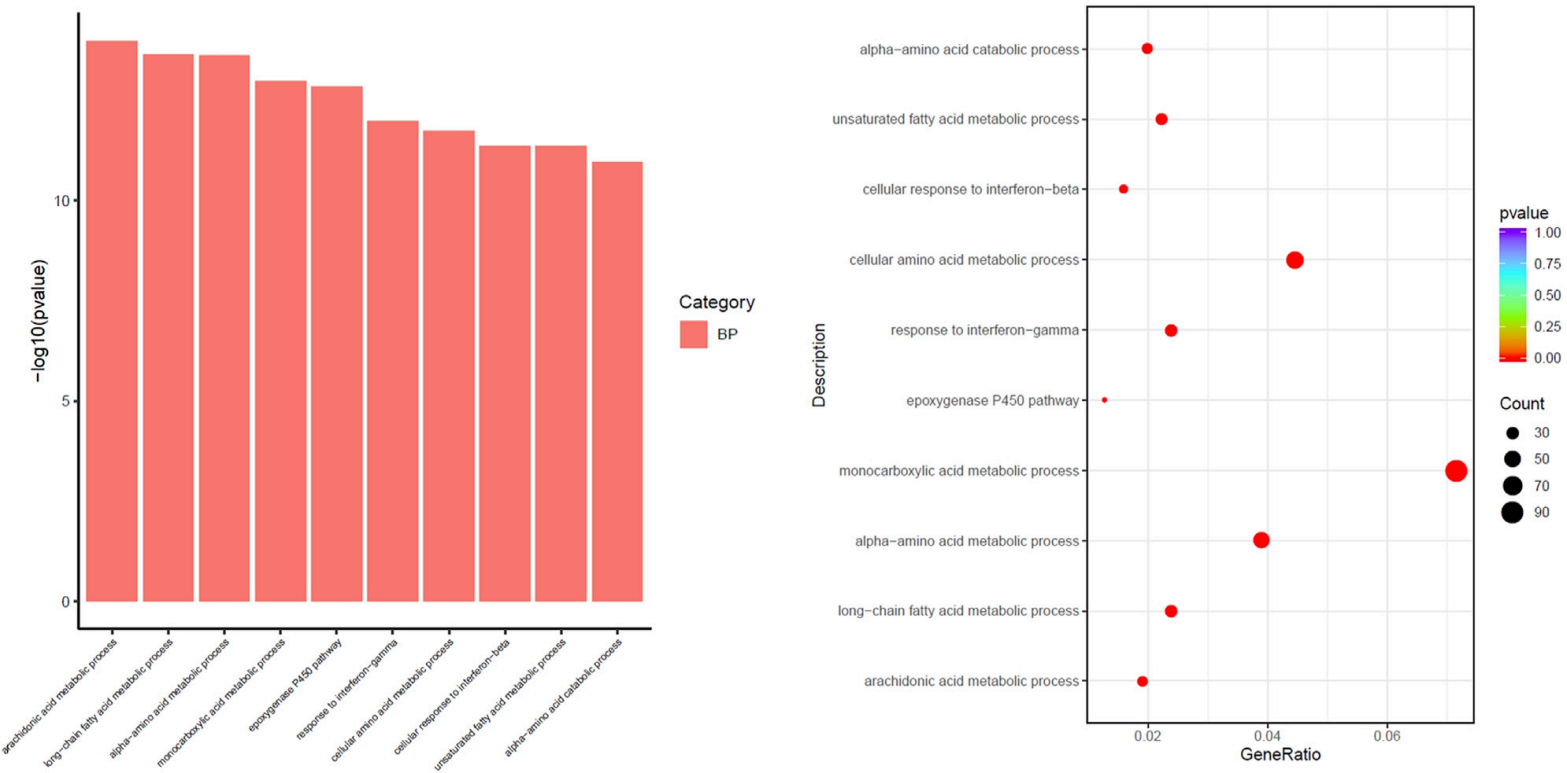
Different biological process of upregulation in the group with PDR syndrome compared with the group with SKYD syndrome.

The differential pathways that were upregulated in the PDR group compared to the SKYD group primarily included the arachidonic acid (AA) metabolic process, epoxygenase P450 pathway, response to interferon-gamma, and cellular response to interferon-beta, as illustrated in Figure 4.

**FIGURE 5:**
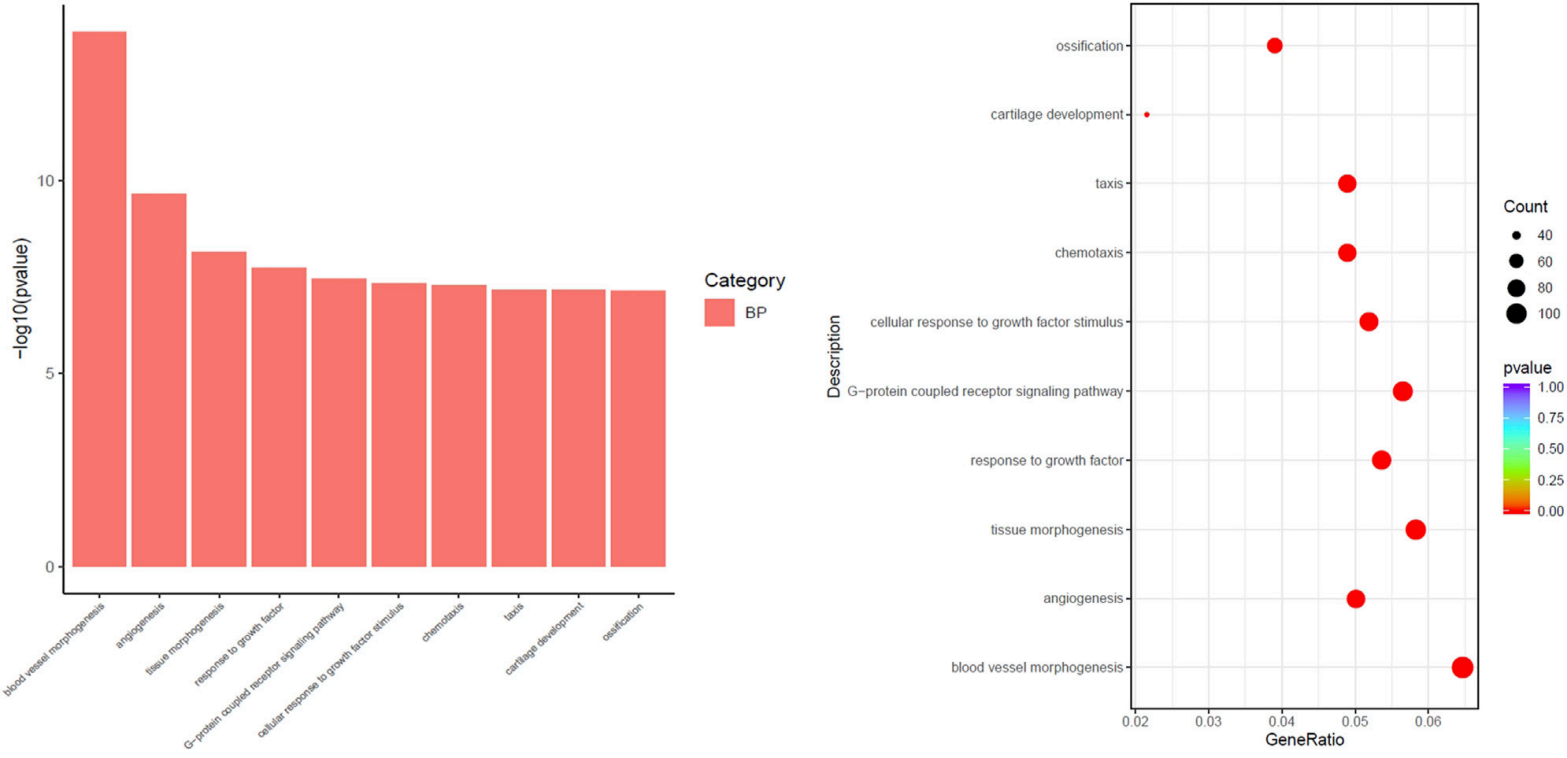
Different biological process of upregulation in the group with SKYD syndrome compared with the group with PDR syndrome.

The differential pathways that were upregulated in the SKYD group compared to the PDR group primarily included blood vessel morphogenesis, angiogenesis, response to growth factor, cellular response to growth factor stimulus, chemotaxis, and taxis. See Figure 5.

## Discussion

Dyslipidemia may significantly increase the morbidity and mortality associated with cardiovascular diseases (Lee et al., 2017). Prior research has found that effective control of blood lipid levels may reduce the possibility of relapsed coronary heart disease and its mortality (Mozaffarian et al., 2015). Therefore, proactive diagnosis and treatment for dyslipidemia, the most critical risk factor of atherosclerosis, is essential for decreasing the incidence and mortality of coronary heart disease and cerebral infarction (Rhee et al., 2019).

Treatment based on syndrome differentiation is the quintessence of TCM (Xu et al., 2021). Syndromes as a distinctive concept in TCM are the core content of the theory and provide vital evidence for treatment and prescription. TCM treatment has been shown to offer advantages in the management of dyslipidemia (Sham et al., 2014; Xu et al., 2019). The predominant mechanisms encompass inhibiting cholesterol absorption in the intestines and biological synthesis of endogenous lipids, regulating lipoprotein lipase activity and cholesterol transport, promoting the conversion of cholesterol into bile acids and cholesterol emission, and the regulatory effects of lipid metabolism-related transcription factors in TCM drugs (Bei et al., 2012). Syndromes represent conclusions made regarding the location and nature of the current stage of a disease, focusing on the pathological changes of the body’s response state at a certain stage of a disease. In the context of the current study, the precise discrimination of exact TCM syndromes of dyslipidemia is the prerequisite and basis of TCM treatment. Therefore, TCM syndrome research is a critical link in TCM modernization, wherein biological syndrome research is most significant, providing evidence for the onset and evolution of syndromes and effective mechanisms behind specific interventions for syndromes.

RNA-Seq is a next-generation approach using high throughput sequencing available for low-abundance genomes. It is sensitive for gene structure analysis and gene expression and function assessments to unveil internal molecular mechanisms behind specific biological processes and the pathogenesis of diseases. The features of these genes consist of classic genetic features (nucleotide sequence changes) and epigenetic features (heritable phenotype changes without nucleotide sequence alterations). Transcriptomics can provide gene expression information under certain conditions and reveal the mechanism of action of specific genes, which is consistent with the TCM theory that diseases express different syndromes under the influence of internal and external causes. Our study investigated the syndrome–biological mechanism of dyslipidemia using RNA-Seq.

Our previous serum metabonomics studies of dyslipidemic patients with PDR syndrome and SKYD syndrome found that the accumulation of harmful metabolites is the predominant metabolic trait in patients with PDR syndrome, while a lack of protective metabolites is the main metabolic feature in patients with SKYD syndrome. Further analysis has shown that oxidation and inflammatory responses are essential contributors to the different metabolic characteristics between the two syndromes (Chen et al., 2019). Thus, in-depth study of SKYD syndrome and PDR syndrome in dyslipidemia necessitates research concerning oxidation and inflammatory responses.

Macrophages are pivotal in the formation of atherosclerosis following oxidative stress damage to the vascular endothelium. M1 macrophages can promote inflammation and tissue damage, while M2 macrophages can release anti-inflammatory factors and enhance plaque stability. Endothelial dysfunction and the subsequent oxidative inflammatory reaction represent the core pathological mechanism of dyslipidemia. Macrophages are recruited toward endothelial cells, which is an early stimulus of atheromatous plaque formation (Tiwari et al., 2008) and the chief basis of the incidence of atherosclerosis. Based on our previous research, in the present study, we established the disease–syndrome animal models for and explored the characteristics of the aortic endothelial macrophages between PDR syndrome and SKYD syndrome of dyslipidemia, using RNA-Seq. Our findings from the current research are presented in the following subsections.

### Quality Assessment of the Dyslipidemia Disease Model Using Serum Lipids Analysis and HE Staining of the Aorta Revealed Success in Disease Modeling

Compared with the NC group, TG, TC, and LDL-C levels significantly increased in mice of the PDR and SKYD groups and exceeded the upper limit of the normal range, in conformity to the diagnostic criterion of dyslipidemia. The HE staining of the aorta revealed a smooth endothelial surface and clear borders between inner, medial, and outer layers in mice from the PDR and SKYD groups, without pronounced lipid accumulation and fatty streaks. These results indicated that there were no pathological manifestations of atherosclerosis in the mice of the two groups. The HE staining results implied the satisfactory quality of the dyslipidemia modeling.

However, there were no significant differences in TG, TC, HDL-C, and LDL-C levels between the PDR and SKYD groups. Therefore, the differences in transcriptomic traits and biological results between the two groups based on RNA-Seq in subsequent studies may not be caused by the disease itself, but by syndromes (subtypes).

### Quality Assessment of the Two Syndrome Models (PDR, SKYD) Through Behavioral Tests Indicated the Feasibility of Syndrome Modeling

The characteristics of mice in the PDR group included their being fat in body shape, with a reduced brightness of hair, lethargy, slow responses, lazy to move, as well as soft, formed, and sticky feces, consistent with clinical manifestations of PDR syndrome. The behavioral features of mice in the SKYD group incorporated matted hair, slight paw and nail coloration, shrinking of the body, tranquil, fatigue, sleepiness, sticking together, low-temperature tail, decrease in food and water intake, and loose and watery stools. These were in agreement with clinical manifestations of SKYD syndrome. These results indicate that syndrome modeling can simulate typical symptoms of the corresponding syndrome.

### Transcriptomic Data of Macrophages Showed Pro-Inflammatory Activities in the Vascular Endothelium in Dyslipidemic Mice With PDR Syndrome and SKYD Syndrome, but the Mechanisms Were Different

#### IFN-γ and IFN-β Expressions Were Upregulated in Macrophages in Dyslipidemic Mice With PDR Syndrome, Promoting Endothelial Inflammation

It is known that interferon gamma (IFN-γ) can facilitate the progression of inflammatory diseases—for example, inflammatory bowel disease and atherosclerosis. *In-vitro* and *in vivo* studies have found IFN-γ may damage epithelial cells and endothelial barrier integrity (Ng et al., 2015). Moreover, IFN-γ exerts significant impacts on the biological properties of the vascular endothelial cells. It may initiate vascular remodeling around microvascular endothelial cells (Chrobak et al., 2013). Monocyte-derived macrophages are induced by the pro-inflammatory factor IFN-γ, which are of vital significance during plaque formation. Both IFN-γ and macrophages are major players in oxidative stress. Just like other pro-inflammatory factors, IFN-γ acts as a significant trigger of the synthesis and release of reactive oxygen species (Schroecksnadel et al., 2006).

Interferon beta (IFN-β) mRNA can effectively induce endothelial chemokine expression (Buttmann et al., 2007). It enhances endothelial cell adhesion to eosinophils mainly through upregulating vascular cell adhesion molecule-1 and intercellular adhesion molecule-1 expressions (Kobayashi et al., 2008). IFN-β fuels the formation of macrophage foam cells via a macrophage scavenger receptor class A (SR-A)-mediated cholesterol influx and an ATP-binding cassette transporter (ABCA1)-mediated efflux of mechanisms, thus expediting the incidence of atherosclerosis (Boshuizen et al., 2016).

Under TCM, PDR syndrome is a type of sthenia syndrome. The phlegm evil is both the pathological product and pathogenic factor, impeding the delivery and movement of Qi, thus resulting in body fluid stagnation and hydrops or damp evil accumulation and phlegm. Turbid phlegm can block the vessels and bring about damage that causes blood vessels to be blocked. This then affects the operation of Qi and blood, resulting in their poor operation, which can lead to the occurrence of diseases over time. Finally, the vascular endothelium is impaired.

Our results showed that IFN-γ and IFN-β expressions in macrophages were upregulated in dyslipidemic mice with PDR syndrome, and at significantly higher levels than in mice with SKYD syndrome. This finding suggests that vascular endothelial injury induced by IFN-γ and IFN-β overexpression in vascular endothelial macrophages is a characteristic of PDR syndrome in dyslipidemia.

#### Macrophage Chemotaxis and Taxis Were Enhanced in Dyslipidemic Mice With SKYD Syndrome, Promoting Endothelial Inflammation

Macrophages are inflammatory cells, and their accumulation can stimulate cytokine and chemokine release, initiating immune responses and accelerating plaque formation (Liehn et al., 2006). The secreted chemokines infiltrate atherosclerotic plaques at an early stage (Weber et al., 2008). Despite the role in lipid accumulation, macrophage foam cells also release pro-inflammatory factors and chemokines, further stimulating vascular endothelial cells, fueling vascular endothelial inflammation, and exacerbating the disease (Kleemann et al., 2008). A prior study found that resveratrol could exert a protective effect on the heart via inhibiting endothelial cell migration and monocyte chemotaxis (Cicha et al., 2011).

The TCM mechanism of SKYD syndrome in dyslipidemia refers to spleen–kidney Yang deficiency. The spleen and kidneys are weak, which will cause the loss of Qi and Yang. The deficiency of spleen Yang and kidney Yang can lead to the loss of a warm body. The function of Qi is abnormal, which causes the decrease of its protective effect. Furthermore, as the body lacks warmth from Yang–Qi and corresponding protection, the vascular endothelium can be easily injured.

Our results showed that macrophage chemotaxis and taxis were significantly enhanced in dyslipidemic mice with SKYD syndrome, compared with the PDR mice. This finding suggests that vascular endothelial injury induced by enhanced macrophage chemotaxis and taxis was the main characteristic of the SKYD syndrome in dyslipidemia.

The above results demonstrate that different biological processes resulted in vascular endothelial injury in PDR syndrome and SKYD syndrome with dyslipidemia, indicating different injury mechanisms of the two syndromes. These results also imply that there exist biological bases behind the pathogenesis of TCM syndromes.

### Transcriptomic Data of Macrophages Showed Protection for the Vascular Endothelium in Dyslipidemic Mice With PDR Syndrome and SKYD Syndrome, but the Mechanisms Were Different

#### AA Metabolic Process and Epoxygenase P450 Pathway Levels Increased in Macrophages in Dyslipidemic Mice With PDR Syndrome, Exerting Protection Effects on the Vascular Endothelium

Growing evidence has shown that AA metabolism is crucial in maintaining vascular homeostasis, closely associated with the occurrence and development of cardiovascular diseases (Xue et al., 2015). AA is an amphiphilic compound affecting endothelial cell migration; without the involvement of receptor-specific signaling, it affects endothelial cell metabolism and membrane viscosity (Jensen et al., 2007). AA metabolic pathways are pivotal in platelet activation and gastric damage (Rukoyatkina et al., 2018). Suppressing AA metabolism can further block endothelial cell migration, inducing cell apoptosis (Jantke et al., 2004). A recent study has reported that *Panax notoginseng* saponins combined with aspirin inhibited platelet activity via enhancing AA metabolism (Wang et al., 2021).

Cytochromes P450 (CYP450) refer to a third pathway for AA metabolism (Roman, 2002). CYP450 metabolites of AA in endothelial cells may impact endothelial function. AA is metabolized by CYP450 and cyclooxygenase (COX) into bioactive eicosanoids, exerting vascular protection effects (Spiecker and Liao, 2005). AA is also metabolized by CYP450 and COX into four regioisomeric epoxyeicosatrienoic acids (EETs) used for bioprotection and cardioprotection (Xu et al., 2011). EETs have multiple nutritive functions, including the anti-inflammatory effect, in their cardioprotection (Node et al., 1999). Decreases in EETs expressions may lead to the onset of cardiovascular diseases and endothelial dysfunction (Bellien et al., 2012).

Our analysis showed significant upregulations of AA metabolic process and epoxygenase P450 pathway levels in macrophages in dyslipidemic mice with PDR syndrome, compared to the SKYD mice. These results indicate that endothelial protection from macrophages *via* AA and CYP450 overexpressions is another trait of PDR syndrome in dyslipidemia.

#### Biological Process Items, Including Angiogenesis, Blood Vessel Morphogenesis, Response to Growth Factor, and Cellular Response to Growth Factor Stimulus, Whose Activities Were Significantly Enhanced for Macrophages in Dyslipidemic Mice With SKYD Syndrome, Which Facilitated Angiogenesis and Vascular Repair

Angiogenesis consists of multiple, intricate, highly coordinated processes in which endothelial cells with dynamic changes are of great importance (Potente et al., 2011). Angiogenesis may occur in the pathological environment (Alvarez Arroyo et al., 1998) and be initiated by endothelial cell activation. The genetic program of endothelial cells triggers the modulation of angiogenic phenotype. Macrophages are significant regulators for tissue homeostasis, growth and repair, and morphogenesis. The growing endothelial cells can respond to extracellular signaling molecules, such as extracellular matrix molecules, chemokines, growth factors, and cell adhesion molecules (Jeong et al., 2017). Growth factors and cytokines secreted from macrophages (Lucas et al., 2010) may promote the formation of new blood vessels via recruiting new blood vessels and modifying the extracellular matrix (Corliss et al., 2016). Vascular endothelial growth factors (VEGFs) are considered the most robust booster for angiogenesis, increasing the survival of endothelial cells and enhancing mitosis (Dvorak, 2005). Numerous studies have demonstrated that VEGFs are expressed in macrophages (Guo et al., 2018). They can stimulate assorted cell functions of endothelial cells via high-affinity binding to two tyrosine kinase receptors, VEGF receptor (VEGFR) 1 and VEGFR2 (Zachary, 2003).

Our study found that the top biological process items enriched in macrophages in dyslipidemic mice with SKYD syndrome were angiogenesis, blood vessel morphogenesis, response to growth factor, and cellular response to growth factor stimulus, whose activities were significantly enhanced, compared to the PDR group. This finding suggested that angiogenesis and vascular repair via enhancing macrophage chemotaxis and taxis are another critical feature of SKYD syndrome in dyslipidemia.

The above results indicate that vascular protection mechanisms are distinct between PDR syndrome and SKYD syndrome in dyslipidemia. This further implies different protection mechanisms in the two syndromes, which in turn further corroborates that there exist biological bases behind the pathogeneses of TCM syndromes.

According to the results presented in Sections *Transcriptomic Data of Macrophages Showed Pro-Inflammatory Activities in the Vascular Endothelium in Dyslipidemic Mice With PDR Syndrome and SKYD Syndrome, but the Mechanisms Were Different* and *Transcriptomic Data of Macrophages Showed Protection for the Vascular Endothelium in Dyslipidemic Mice With PDR Syndrome and SKYD Syndrome, but the Mechanisms Were Different*, vascular endothelial injury was induced by different biological processes in PDR syndrome versus SKYD syndrome in dyslipidemia. But there was also found to be angiogenesis and vascular repair in the pathogenetic process. The two opposite processes—injury and repair—are consistent with the TCM tenet that “healthy Qi and evil Qi are struggling throughout the incidence and dynamic development of diseases and syndromes.” As the injury effect outweighs the repairing effect, the state of diseases takes place.

### Different Transcriptomic Characteristics of Aortic Endothelial Macrophages Between Dyslipidemic Mice With PDR Syndrome and SKYD Syndrome are Manifested by Distinct Biological Control Processes During Both Harmful and Protective Biological Processes, Indicating Different Biological Bases Behind Different Syndromes of the Same Disease and Providing Biological Evidence for the TCM Principle of “Treating the Same Disease With Different Treatments”

See Figure 6.

**FIGURE 6:**
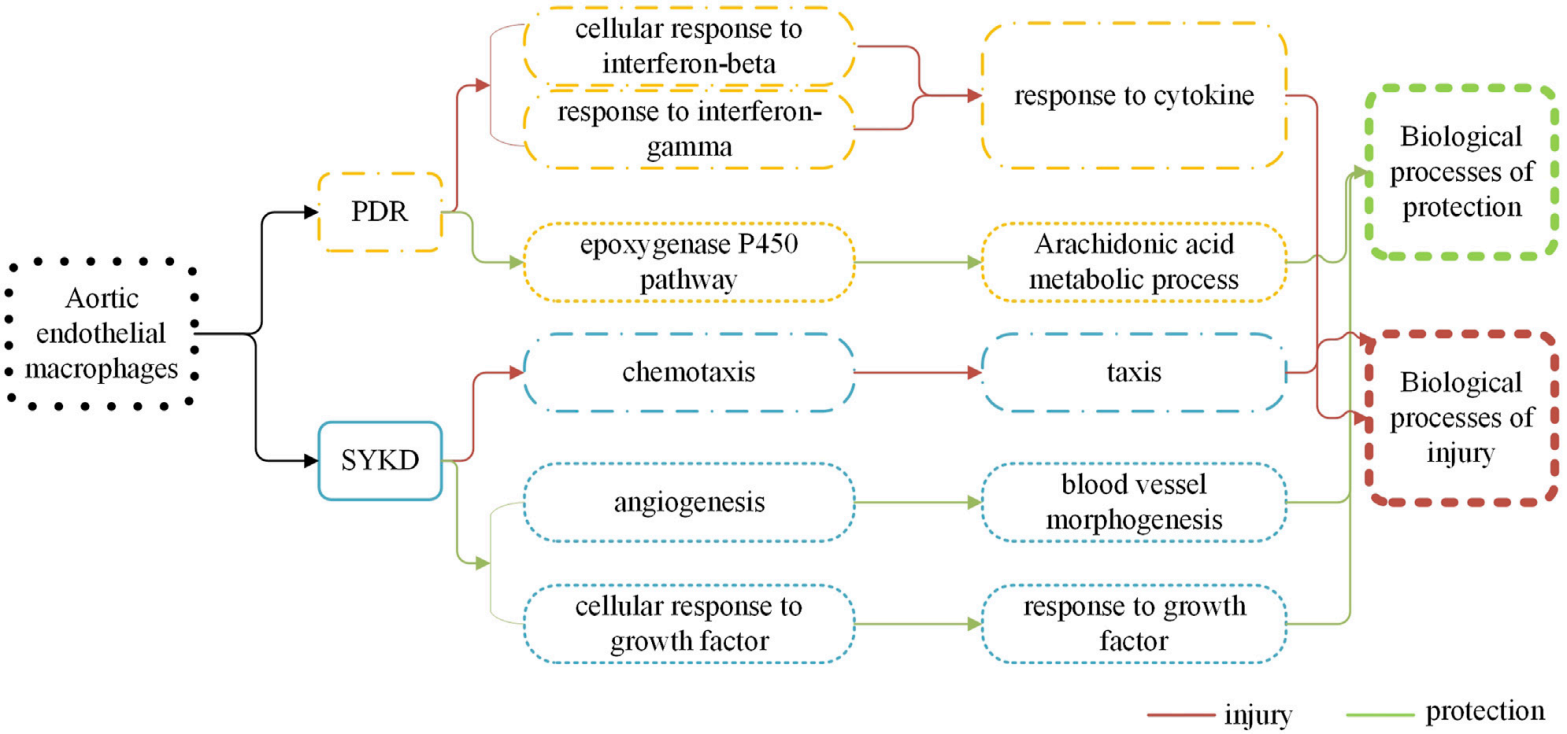
There are different biological processes of macrophages in the dyslipidemia PDR syndrome group and the SKYD group.

In the TCM clinic, Western medicine diagnosis is often combined with TCM syndrome diagnosis for the management of a disease. Research about the association between Western diseases and TCM syndromes is believed to be one of the most important steps for modern TCM diagnostics studies. Based on the confirmation of a Western disease, disease–syndrome research can not only elucidate the biological bases for TCM differentiation but also help to advance innovative research on the “disease–syndrome therapy formula” and thereby provide precise and rational treatment.

Previous TCM syndrome research has emphasized harmful factors in a syndrome scenario, without much attention to the body’s self-protection in this process. The present study, however, did both, focusing on an analysis of harmful factors and protective factors, and thereby providing an innovative dimension. The unity of opposites—protective and harmful effects—is achieved through dynamic balancing between healthy Qi and evil Qi via mutual conflicts, mutual restriction, and mutual repulsion. Diseases can occur when evil Qi outstrips healthy Qi, per the TCM principle that affirms, “when there is sufficient healthy Qi inside, pathogenic factors have no way to invade the body; where pathogenic factors accumulate, the parts of the body must be deficient in the healthy Qi.” TCM treatment should be implemented based on the accurate discrimination of diseases and syndromes and precisely resolve issues by correcting an imbalance between healthy Qi and evil Qi through prescriptions and formulas.

Our transcriptomic analysis of aortic endothelial macrophages in dyslipidemic mice with PDR syndrome and SKYD syndrome provided notable results, as follows. First, DEGs were identified between dyslipidemic mice with PDR syndrome versus SKYD syndrome, proving different biological mechanisms during the pathogenesis of different syndromes, from the perspective of syndrome research. Second, there were found to exist different biological processes between PDR syndrome and SKYD syndrome in dyslipidemic patients, including harmful and protective biological processes. When evil Qi invades the body to produce harmful effects, healthy Qi also responds to it and thereby generates protective responses in a syndrome scenario, which corresponds with the TCM principle that “healthy Qi and evil Qi are struggling throughout the incidence and dynamic development of diseases and syndromes.” Therefore, the occurrence of syndromes can be understood to be a result of healthy energy–evil struggles. This, in turn, may help to explain why TCM treatment for one disease with various therapies and formulas can achieve satisfactory efficacy, in that it could be attributed to different drugs targeting different biological processes. Our work offers biological mechanisms for the TCM theories “treating different syndromes with different treatments” and “formula corresponding to the syndrome.” Third, though patients may be diagnosed with the same disease (i.e., dyslipidemia), different formulas should be selected according to their particular syndromes (i.e., giving consideration to the distinct biological processes occurring within PDR syndrome vs. SKYD syndrome). Our study provides biological evidence behind the TCM principle of “treating the same disease with different treatments,” embodying the scientificity of “treatment based on syndrome differentiation.”

## Data Availability

The datasets presented in this study can be found in online repositories. The names of the repository/repositories and accession number(s) can be found below: NCBI [accession: https://www.ncbi.nlm.nih.gov/geo/query/acc.cgi?acc=GSE180802].

## References

[B1] Alvarez Arroyo M. V. Caramelo C. Angeles Castilla M. González Pacheco F. R. Martín O. Arias J. (1998). Role of Vascular Endothelial Growth Factor in the Response to Vessel Injury. Kidney Int. Suppl. 68, S7–S9. 10.1046/j.1523-1755.1998.06804.x 9839275

[B2] Bei W. J. Guo J. Wu H. Y. Cao Y. (2012). Lipid-regulating Effect of Traditional Chinese Medicine: Mechanisms of Actions. Evid. Based Complement. Alternat Med. 2012, 970635. 10.1155/2012/970635 22611438 PMC3352575

[B3] Bellien J. Iacob M. Remy-Jouet I. Lucas D. Monteil C. Gutierrez L. (2012). Epoxyeicosatrienoic Acids Contribute with Altered Nitric Oxide and Endothelin-1 Pathways to Conduit Artery Endothelial Dysfunction in Essential Hypertension. Circulation 125 (10), 1266–1275. 10.1161/CIRCULATIONAHA.111.070680 22412088

[B4] Boshuizen M. C. Hoeksema M. A. Neele A. E. van der Velden S. Hamers A. A. Van den Bossche J. (2016). Interferon-β Promotes Macrophage Foam Cell Formation by Altering Both Cholesterol Influx and Efflux Mechanisms. Cytokine 77, 220–226. 10.1016/j.cyto.2015.09.016 26427927

[B5] Buttmann M. Berberich-Siebelt F. Serfling E. Rieckmann P. (2007). Interferon-beta Is a Potent Inducer of Interferon Regulatory Factor-1/2-dependent IP-10/CXCL10 Expression in Primary Human Endothelial Cells. J. Vasc. Res. 44 (1), 51–60. 10.1159/000097977 17167270

[B6] Chen J. Ye C. Hu X. Huang C. Yang Z. Li P. (2019). Serum Metabolomics Model and its Metabolic Characteristics in Patients with Different Syndromes of Dyslipidemia Based on Nuclear Magnetic Resonance. J. Pharm. Biomed. Anal. 167, 100–113. 10.1016/j.jpba.2018.12.042 30763881

[B7] Chen J. Ye C. Yang Z. Xue X. Sun Q. Li P. (2016). The Correlation between the Traditional Chinese Medicine (TCM) Syndrome and the Concentration of Adiponectin and Peroxynitrite in Dyslipidemia Patients. Eur. J. Integr. Med. 8 (6), 973–979. 10.1016/j.eujim.2016.04.009

[B8] Chrobak I. Lenna S. Stawski L. Trojanowska M. (2013). Interferon-γ Promotes Vascular Remodeling in Human Microvascular Endothelial Cells by Upregulating Endothelin (ET)-1 and Transforming Growth Factor (TGF) β2. J. Cel Physiol 228 (8), 1774–1783. 10.1002/jcp.24337 PMC4072032 23359533

[B9] Chu J. G. R. Zhao S. (2016). Guidelines for the Prevention and Treatment of Dyslipidemia in Chinese Adults (Revised Edition in 2016). Chin. Circ. J. 31 (10), 937–953.

[B10] Ciccone M. M. Miniello V. Marchioli R. Scicchitano P. Cortese F. Palumbo V. (2011). Morphological and Functional Vascular Changes Induced by Childhood Obesity. Eur. J. Cardiovasc. Prev. Rehabil. 18 (6), 831–835. 10.1177/1741826711398180 21450599

[B11] Cicha I. Regler M. Urschel K. Goppelt-Struebe M. Daniel W. G. Garlichs C. D. (2011). Resveratrol Inhibits Monocytic Cell Chemotaxis to MCP-1 and Prevents Spontaneous Endothelial Cell Migration through Rho Kinase-dependent Mechanism. J. Atheroscler. Thromb. 18 (12), 1031–1042. 10.5551/jat.8136 21878744

[B12] Corliss B. A. Azimi M. S. Munson J. M. Peirce S. M. Murfee W. L. (2016). Macrophages: An Inflammatory Link between Angiogenesis and Lymphangiogenesis. Microcirculation 23 (2), 95–121. 10.1111/micc.12259 26614117 PMC4744134

[B13] Davies L. C. Taylor P. R. (2015). Tissue-resident Macrophages: Then and Now. Immunology 144 (4), 541–548. 10.1111/imm.12451 25684236 PMC4368161

[B14] Dvorak H. F. (2005). Angiogenesis: Update 2005. J. Thromb. Haemost. 3 (8), 1835–1842. 10.1111/j.1538-7836.2005.01361.x 16102050

[B15] Eelen G. Treps L. Li X. Carmeliet P. (2020). Basic and Therapeutic Aspects of Angiogenesis Updated. Circ. Res. 127 (2), 310–329. 10.1161/CIRCRESAHA.120.316851 32833569

[B16] Gan L. Jiang T. T. Yi W. J. Lu R. Xu F. Y. Liu C. M. (2020). Study on Potential Biomarkers of Energy Metabolism-Related to Early-Stage Yin-Deficiency-Heat Syndrome Based on Metabolomics and Transcriptomics. Anat. Rec. (Hoboken) 303 (8), 2109–2120. 10.1002/ar.24355 31909898

[B17] Guo L. Akahori H. Harari E. Smith S. L. Polavarapu R. Karmali V. (2018). CD163+ Macrophages Promote Angiogenesis and Vascular Permeability Accompanied by Inflammation in Atherosclerosis. J. Clin. Invest. 128 (3), 1106–1124. 10.1172/JCI93025 29457790 PMC5824873

[B18] He C. Fu P. Zhang K. Xia Q. Yang Y. Xie L. (2018). Chinese Herbal Medicine for Dyslipidemia: Protocol for a Systematic Review and Meta-Analysis. Medicine (Baltimore) 97 (44), e13048. 10.1097/MD.0000000000013048 30383674 PMC6221744

[B19] Hu Y. Ehli E. A. Kittelsrud J. Ronan P. J. Munger K. Downey T. (2012). Lipid-lowering Effect of Berberine in Human Subjects and Rats. Phytomedicine 19 (10), 861–867. 10.1016/j.phymed.2012.05.009 22739410

[B20] Jantke J. Ladehoff M. Kürzel F. Zapf S. Kim E. Giese A. (2004). Inhibition of the Arachidonic Acid Metabolism Blocks Endothelial Cell Migration and Induces Apoptosis. Acta Neurochir (Wien) 146 (5), 483–494. 10.1007/s00701-004-0238-z 15118886

[B21] Jensen L. D. Hansen A. J. Lundbaek J. A. (2007). Regulation of Endothelial Cell Migration by Amphiphiles - Are Changes in Cell Membrane Physical Properties Involved?. Angiogenesis 10 (1), 13–22. 10.1007/s10456-006-9060-y 17265099

[B22] Jeong H. W. Hernández-Rodríguez B. Kim J. Kim K. P. Enriquez-Gasca R. Yoon J. (2017). Transcriptional Regulation of Endothelial Cell Behavior during Sprouting Angiogenesis. Nat. Commun. 8 (1), 726. 10.1038/s41467-017-00738-7 28959057 PMC5620061

[B23] Jiang T. T. Li J. C. (2020). Review on the Systems Biology Research of Yin‐deficiency‐heat Syndrome in Traditional Chinese Medicine. Anat. Rec., 1–6. 10.1002/ar.24354 31909899

[B24] Kleemann R. Zadelaar S. Kooistra T. (2008). Cytokines and Atherosclerosis: a Comprehensive Review of Studies in Mice. Cardiovasc. Res. 79 (3), 360–376. 10.1093/cvr/cvn120 18487233 PMC2492729

[B25] Kobayashi T. Takaku Y. Yokote A. Miyazawa H. Soma T. Hagiwara K. (2008). Interferon-beta Augments Eosinophil Adhesion-Inducing Activity of Endothelial Cells. Eur. Respir. J. 32 (6), 1540–1547. 10.1183/09031936.00059507 18653650

[B26] Lee J. S. Chang P. Y. Zhang Y. Kizer J. R. Best L. G. Howard B. V. (2017). Triglyceride and HDL-C Dyslipidemia and Risks of Coronary Heart Disease and Ischemic Stroke by Glycemic Dysregulation Status: The Strong Heart Study. Diabetes Care 40 (4), 529–537. 10.2337/dc16-1958 28122840 PMC5360283

[B27] Li Z. Zhao T. Tan X. Lei S. Huang L. Yang L. (2019). Polymorphisms in PCSK9, LDLR, BCMO1, SLC12A3, and KCNJ1 Are Associated with Serum Lipid Profile in Chinese Han Population. Int. J. Environ. Res. Public Health 16 (17), 3207. 10.3390/ijerph16173207 PMC6747169 31480784

[B28] Liehn E. A. Zernecke A. Postea O. Weber C. (2006). Chemokines: Inflammatory Mediators of Atherosclerosis. Arch. Physiol. Biochem. 112 (4-5), 229–238. 10.1080/13813450601093583 17178596

[B29] Lin C. C. Li T. C. Lai M. M. (2005). Efficacy and Safety of Monascus purpureus Went rice in Subjects with Hyperlipidemia. Eur. J. Endocrinol. 153 (5), 679–686. 10.1530/eje.1.02012 16260426

[B30] Lucas T. Waisman A. Ranjan R. Roes J. Krieg T. Müller W. (2010). Differential Roles of Macrophages in Diverse Phases of Skin Repair. J. Immunol. 184 (7), 3964–3977. 10.4049/jimmunol.0903356 20176743

[B31] Moore E. M. West J. L. (2019). Harnessing Macrophages for Vascularization in Tissue Engineering. Ann. Biomed. Eng. 47 (2), 354–365. 10.1007/s10439-018-02170-4 30535815

[B32] Mozaffarian D. Benjamin E. J. Go A. S. Arnett D. K. Blaha M. J. Cushman M. (2015). Heart Disease and Stroke Statistics--2015 Update: a Report from the American Heart Association. Circulation 131 (4), e29–322. 10.1161/CIR.0000000000000152 25520374

[B33] Ng C. T. Fong L. Y. Sulaiman M. R. Moklas M. A. Yong Y. K. Hakim M. N. (2015). Interferon-Gamma Increases Endothelial Permeability by Causing Activation of P38 MAP Kinase and Actin Cytoskeleton Alteration. J. Interferon Cytokine Res. 35 (7), 513–522. 10.1089/jir.2014.0188 25830506

[B34] Node K. Huo Y. Ruan X. Yang B. Spiecker M. Ley K. (1999). Anti-inflammatory Properties of Cytochrome P450 Epoxygenase-Derived Eicosanoids. Science 285 (5431), 1276–1279. 10.1126/science.285.5431.1276 10455056 PMC2720027

[B35] Pantos J. Efstathopoulos E. Katritsis D. G. (2007). Vascular wall Shear Stress in Clinical Practice. Curr. Vasc. Pharmacol. 5 (2), 113–119. 10.2174/157016107780368253 17430215

[B36] Pesce J. T. Ramalingam T. R. Mentink-Kane M. M. Wilson M. S. El Kasmi K. C. Smith A. M. (2009). Arginase-1-expressing Macrophages Suppress Th2 Cytokine-Driven Inflammation and Fibrosis. Plos Pathog. 5 (4), e1000371. 10.1371/journal.ppat.1000371 19360123 PMC2660425

[B37] Potente M. Gerhardt H. Carmeliet P. (2011). Basic and Therapeutic Aspects of Angiogenesis. Cell 146 (6), 873–887. 10.1016/j.cell.2011.08.039 21925313

[B38] Rhee E. J. Kim H. C. Kim J. H. Lee E. Y. Kim B. J. Kim E. M. (2019). 2018 Guidelines for the Management of Dyslipidemia in Korea. Korean J. Intern. Med. 34 (5), 1171. 10.3904/kjim.2019.188.e1 31466435 PMC6718745

[B39] Roman R. J. (2002). P-450 Metabolites of Arachidonic Acid in the Control of Cardiovascular Function. Physiol. Rev. 82 (1), 131–185. 10.1152/physrev.00021.2001 11773611

[B40] Rukoyatkina N. Shpakova V. Panteleev M. Kharazova A. Gambaryan S. Geiger J. (2018). Multifaceted Effects of Arachidonic Acid and Interaction with Cyclic Nucleotides in Human Platelets. Thromb. Res. 171, 22–30. 10.1016/j.thromres.2018.09.047 30240944

[B41] Sarzynski M. A. Schuna J. M. Jr. Carnethon M. R. Jacobs D. R. Jr. Lewis C. E. Quesenberry C. P. Jr. (2015). Association of Fitness with Incident Dyslipidemias over 25 Years in the Coronary Artery Risk Development in Young Adults Study. Am. J. Prev. Med. 49 (5), 745–752. 10.1016/j.amepre.2015.04.022 26165197 PMC4615297

[B42] Schroecksnadel K. Frick B. Winkler C. Fuchs D. (2006). Crucial Role of Interferon-Gamma and Stimulated Macrophages in Cardiovascular Disease. Curr. Vasc. Pharmacol. 4 (3), 205–213. 10.2174/157016106777698379 16842138

[B43] Scicchitano P. Cortese F. Gesualdo M. De Palo M. Massari F. Giordano P. (2019). The Role of Endothelial Dysfunction and Oxidative Stress in Cerebrovascular Diseases. Free Radic. Res. 53 (6), 579–595. 10.1080/10715762.2019.1620939 31106620

[B44] Sham T. T. Chan C. O. Wang Y. H. Yang J. M. Mok D. K. Chan S. W. (2014). A Review on the Traditional Chinese Medicinal Herbs and Formulae with Hypolipidemic Effect. Biomed. Res. Int. 2014, 925302. 10.1155/2014/925302 25110708 PMC4109135

[B45] Spiecker M. Liao J. K. (2005). Vascular Protective Effects of Cytochrome P450 Epoxygenase-Derived Eicosanoids. Arch. Biochem. Biophys. 433 (2), 413–420. 10.1016/j.abb.2004.10.009 15581597

[B46] Stone N. J. Robinson J. G. Lichtenstein A. H. Bairey Merz C. N. Blum C. B. Eckel R. H. (2014). 2013 ACC/AHA Guideline on the Treatment of Blood Cholesterol to Reduce Atherosclerotic Cardiovascular Risk in Adults: a Report of the American College of Cardiology/American Heart Association Task Force on Practice Guidelines. Circulation 129, S1–S45. 10.1161/01.cir.0000437738.63853.7a 24222016

[B47] Tiwari R. L. Singh V. Barthwal M. K. (2008). Macrophages: an Elusive yet Emerging Therapeutic Target of Atherosclerosis. Med. Res. Rev. 28 (4), 483–544. 10.1002/med.20118 18000963

[B48] Wang W. Yang L. Song L. Guo M. Li C. Yang B. (2021). Combination of Panax Notoginseng Saponins and Aspirin Potentiates Platelet Inhibition with Alleviated Gastric Injury via Modulating Arachidonic Acid Metabolism. Biomed. Pharmacother. 134, 111165. 10.1016/j.biopha.2020.111165 33370633

[B49] Weber C. Zernecke A. Libby P. (2008). The Multifaceted Contributions of Leukocyte Subsets to Atherosclerosis: Lessons from Mouse Models. Nat. Rev. Immunol. 8 (10), 802–815. 10.1038/nri2415 18825131

[B50] Xu X. Zhang X. A. Wang D. W. (2011). The Roles of CYP450 Epoxygenases and Metabolites, Epoxyeicosatrienoic Acids, in Cardiovascular and Malignant Diseases. Adv. Drug Deliv. Rev. 63 (8), 597–609. 10.1016/j.addr.2011.03.006 21477627

[B54] Xu H. Y. Zhang Y. Q. Liu Z. M. Chen T. Lv C. Y. Tang S. H. (2019). ETCM: An Encyclopaedia of Traditional Chinese Medicine. Nucl. Acids Res. 47 (D1), D976–D982. 10.1093/nar/gky987 30365030 PMC6323948

[B55] Xu H. Zhang Y. Wang P. Zhang J. Chen H. Zhang L. (2021). A Comprehensive Review of Integrative Pharmacology-Based Investigation: A Paradigm Shift in Traditional Chinese medicine. Acta Pharm. Sin B 11 (6), 1379–1399. 10.1016/j.apsb.2021.03.024 34221858 PMC8245857

[B51] Xue S. S. He J. L. Zhang X. Liu Y. J. Xue F. X. Wang C. J. (2015). Metabolomic Analysis Revealed the Role of DNA Methylation in the Balance of Arachidonic Acid Metabolism and Endothelial Activation. Biochim. Biophys. Acta 1851 (10), 1317–1326. 10.1016/j.bbalip.2015.07.001 26170200

[B52] Yamada Y. Doi T. Hamakubo T. Kodama T. (1998). Scavenger Receptor Family Proteins: Roles for Atherosclerosis, Host Defence and Disorders of the central Nervous System. Cell Mol Life Sci 54 (7), 628–640. 10.1007/s000180050191 9711230 PMC11147329

[B53] Zachary I. (2003). VEGF Signalling: Integration and Multi-Tasking in Endothelial Cell Biology. Biochem. Soc. Trans. 31 (Pt 6), 1171–1177. 10.1042/bst0311171 14641020

