# Supplementary material for: Study on the Effect of Macrophages on Vascular Endothelium in Mice With Different TCM Syndromes of Dyslipidemia and its Biological Basis Based on RNA-Seq Technology
Source: Front Pharmacol. 2021 Aug 26;12:665635. doi: 10.3389/fphar.2021.665635 (PMC8427158; doi:10.3389/fphar.2021.665635)
Supplement: Supplementary file 1 [file DataSheet2.docx]

**Supplementary File 2** **Injury or protection of macrophages and vascular endothelium in mice with different syndromes of dyslipidemia related differentially expressed genes**

| **Gene ID** | **PDR_ count** | **SKYD_ count** | ***p* value** |  | **Gene ID** | **PDR_ count** | **SKYD_ count** | ***p* value** |
| --- | --- | --- | --- | --- | --- | --- | --- | --- |
| ENSMUSG00000022445 | 323 | 47 | 0.00 |  | ENSMUSG00000026104 | 1169 | 355 | 0.00 |
| ENSMUSG00000032487 | 57 | 2 | 0.00 |  | ENSMUSG00000035042 | 2000 | 645 | 0.00 |
| ENSMUSG00000025002 | 73 | 8 | 0.00 |  | ENSMUSG00000018930 | 875 | 280 | 0.00 |
| ENSMUSG00000060407 | 456 | 100 | 0.00 |  | ENSMUSG00000035373 | 2723 | 943 | 0.00 |
| ENSMUSG00000068086 | 866 | 203 | 0.00 |  | ENSMUSG00000055170 | 39 | 6 | 0.00 |
| ENSMUSG00000025479 | 3178 | 776 | 0.00 |  | ENSMUSG00000022504 | 1244 | 432 | 0.00 |
| ENSMUSG00000060613 | 1120 | 279 | 0.00 |  | ENSMUSG00000024411 | 16 | 0 | 0.00 |
| ENSMUSG00000052974 | 706 | 175 | 0.00 |  | ENSMUSG00000029298 | 686 | 256 | 0.00 |
| ENSMUSG00000094806 | 514 | 128 | 0.00 |  | ENSMUSG00000041515 | 5566 | 2360 | 0.00 |
| ENSMUSG00000003053 | 3283 | 926 | 0.00 |  | ENSMUSG00000074151 | 241 | 96 | 0.00 |
| ENSMUSG00000025197 | 100 | 20 | 0.00 |  | ENSMUSG00000000791 | 204 | 83 | 0.00 |
| ENSMUSG00000054827 | 1406 | 415 | 0.00 |  | ENSMUSG00000049093 | 19 | 3 | 0.00 |
| ENSMUSG00000092008 | 30 | 2 | 0.00 |  | ENSMUSG00000030966 | 800 | 382 | 0.01 |
| ENSMUSG00000030483 | 61 | 13 | 0.00 |  | ENSMUSG00000026866 | 485 | 240 | 0.01 |
| ENSMUSG00000005547 | 252 | 82 | 0.00 |  | ENSMUSG00000054072 | 5433 | 269 | 0.00 |
| ENSMUSG00000033174 | 211 | 67 | 0.00 |  | ENSMUSG00000078921 | 5214 | 719 | 0.00 |
| ENSMUSG00000042248 | 216 | 71 | 0.00 |  | ENSMUSG00000068606 | 130 | 9 | 0.00 |
| ENSMUSG00000068083 | 54 | 13 | 0.00 |  | ENSMUSG00000073555 | 1016 | 177 | 0.00 |
| ENSMUSG00000025004 | 57 | 14 | 0.00 |  | ENSMUSG00000090942 | 146 | 20 | 0.00 |
| ENSMUSG00000074882 | 299 | 123 | 0.00 |  | ENSMUSG00000048852 | 104 | 15 | 0.00 |
| ENSMUSG00000062624 | 200 | 82 | 0.00 |  | ENSMUSG00000046879 | 10788 | 2545 | 0.00 |
| ENSMUSG00000067225 | 324 | 140 | 0.00 |  | ENSMUSG00000074896 | 2248 | 623 | 0.00 |
| ENSMUSG00000024292 | 105 | 40 | 0.00 |  | ENSMUSG00000078920 | 14916 | 4448 | 0.00 |
| ENSMUSG00000061740 | 96 | 40 | 0.01 |  | ENSMUSG00000078853 | 6044 | 1992 | 0.00 |
| ENSMUSG00000057666 | 4375 | 77 | 0.00 |  | ENSMUSG00000058163 | 432 | 208 | 0.01 |
| ENSMUSG00000040264 | 4138 | 114 | 0.00 |  | ENSMUSG00000004814 | 326 | 5501 | 0.00 |
| ENSMUSG00000022126 | 1783 | 66 | 0.00 |  | ENSMUSG00000076431 | 19 | 455 | 0.00 |
| ENSMUSG00000078922 | 3013 | 362 | 0.00 |  | ENSMUSG00000040152 | 392 | 5031 | 0.00 |
| ENSMUSG00000028270 | 7705 | 1058 | 0.00 |  | ENSMUSG00000022309 | 0 | 90 | 0.00 |
| ENSMUSG00000004296 | 121 | 13 | 0.00 |  | ENSMUSG00000031785 | 0 | 88 | 0.00 |
| ENSMUSG00000028268 | 6736 | 1336 | 0.00 |  | ENSMUSG00000036856 | 0 | 77 | 0.00 |
| ENSMUSG00000069874 | 1240 | 268 | 0.00 |  | ENSMUSG00000019997 | 1 | 89 | 0.00 |
| ENSMUSG00000105096 | 93 | 13 | 0.00 |  | ENSMUSG00000018593 | 187 | 1817 | 0.00 |
| ENSMUSG00000104713 | 389 | 83 | 0.00 |  | ENSMUSG00000022018 | 848 | 7779 | 0.00 |
| ENSMUSG00000035385 | 5307 | 1309 | 0.00 |  | ENSMUSG00000022469 | 66 | 674 | 0.00 |
| ENSMUSG00000030895 | 4179 | 1033 | 0.00 |  | ENSMUSG00000061878 | 15 | 217 | 0.00 |
| ENSMUSG00000060550 | 12164 | 3456 | 0.00 |  | ENSMUSG00000031740 | 4 | 98 | 0.00 |
| ENSMUSG00000079363 | 1587 | 447 | 0.00 |  | ENSMUSG00000010660 | 6 | 114 | 0.00 |
| ENSMUSG00000018899 | 5290 | 1555 | 0.00 |  | ENSMUSG00000038545 | 1 | 69 | 0.00 |
| ENSMUSG00000105504 | 237 | 63 | 0.00 |  | ENSMUSG00000006445 | 37 | 359 | 0.00 |

| **Gene ID** | **PDR_ count** | **SKYD_ count** | ***p* value** |  | **Gene ID** | **PDR_ count** | **SKYD_ count** | ***p* value** |
| --- | --- | --- | --- | --- | --- | --- | --- | --- |
| ENSMUSG00000032011 | 149 | 1190 | 0.00 |  | ENSMUSG00000044317 | 0 | 14 | 0.00 |
| ENSMUSG00000004951 | 310 | 2300 | 0.00 |  | ENSMUSG00000003032 | 70 | 229 | 0.00 |
| ENSMUSG00000029373 | 3690 | 26049 | 0.00 |  | ENSMUSG00000018500 | 418 | 1239 | 0.00 |
| ENSMUSG00000024486 | 0 | 53 | 0.00 |  | ENSMUSG00000023031 | 71 | 227 | 0.00 |
| ENSMUSG00000050711 | 0 | 49 | 0.00 |  | ENSMUSG00000019256 | 52 | 170 | 0.00 |
| ENSMUSG00000025355 | 290 | 1978 | 0.00 |  | ENSMUSG00000020063 | 46 | 153 | 0.00 |
| ENSMUSG00000037095 | 2041 | 13391 | 0.00 |  | ENSMUSG00000049130 | 1852 | 5261 | 0.00 |
| ENSMUSG00000037362 | 0 | 44 | 0.00 |  | ENSMUSG00000022836 | 0 | 13 | 0.00 |
| ENSMUSG00000049791 | 24 | 201 | 0.00 |  | ENSMUSG00000010175 | 0 | 13 | 0.00 |
| ENSMUSG00000034353 | 1199 | 6646 | 0.00 |  | ENSMUSG00000045930 | 0 | 13 | 0.00 |
| ENSMUSG00000067336 | 1 | 44 | 0.00 |  | ENSMUSG00000032494 | 0 | 13 | 0.00 |
| ENSMUSG00000037411 | 0 | 33 | 0.00 |  | ENSMUSG00000028195 | 12 | 54 | 0.00 |
| ENSMUSG00000050953 | 0 | 33 | 0.00 |  | ENSMUSG00000005413 | 1251 | 3480 | 0.00 |
| ENSMUSG00000002603 | 72 | 381 | 0.00 |  | ENSMUSG00000054364 | 185 | 535 | 0.00 |
| ENSMUSG00000016494 | 58 | 313 | 0.00 |  | ENSMUSG00000022475 | 96 | 284 | 0.00 |
| ENSMUSG00000044337 | 304 | 1399 | 0.00 |  | ENSMUSG00000026836 | 0 | 12 | 0.00 |
| ENSMUSG00000001300 | 35 | 189 | 0.00 |  | ENSMUSG00000073599 | 10 | 43 | 0.00 |
| ENSMUSG00000019929 | 1375 | 5747 | 0.00 |  | ENSMUSG00000030069 | 4 | 26 | 0.00 |
| ENSMUSG00000004791 | 0 | 24 | 0.00 |  | ENSMUSG00000031565 | 198 | 544 | 0.00 |
| ENSMUSG00000021611 | 0 | 24 | 0.00 |  | ENSMUSG00000017417 | 75 | 214 | 0.00 |
| ENSMUSG00000034881 | 247 | 976 | 0.00 |  | ENSMUSG00000031503 | 2 | 19 | 0.00 |
| ENSMUSG00000028108 | 7728 | 28858 | 0.00 |  | ENSMUSG00000022505 | 2 | 19 | 0.00 |
| ENSMUSG00000038742 | 16 | 89 | 0.00 |  | ENSMUSG00000042745 | 416 | 1089 | 0.00 |
| ENSMUSG00000040289 | 0 | 20 | 0.00 |  | ENSMUSG00000068196 | 0 | 11 | 0.00 |
| ENSMUSG00000025473 | 497 | 1724 | 0.00 |  | ENSMUSG00000042258 | 0 | 11 | 0.00 |
| ENSMUSG00000001827 | 0 | 19 | 0.00 |  | ENSMUSG00000031465 | 0 | 11 | 0.00 |
| ENSMUSG00000031250 | 0 | 18 | 0.00 |  | ENSMUSG00000026883 | 14 | 54 | 0.00 |
| ENSMUSG00000045005 | 0 | 17 | 0.00 |  | ENSMUSG00000033191 | 1 | 14 | 0.00 |
| ENSMUSG00000044562 | 0 | 17 | 0.00 |  | ENSMUSG00000031613 | 877 | 2203 | 0.00 |
| ENSMUSG00000006235 | 0 | 17 | 0.00 |  | ENSMUSG00000024241 | 15 | 55 | 0.00 |
| ENSMUSG00000000392 | 0 | 17 | 0.00 |  | ENSMUSG00000015468 | 11 | 41 | 0.00 |
| ENSMUSG00000020676 | 18 | 84 | 0.00 |  | ENSMUSG00000024087 | 0 | 10 | 0.00 |
| ENSMUSG00000022893 | 0 | 16 | 0.00 |  | ENSMUSG00000055254 | 0 | 10 | 0.00 |
| ENSMUSG00000027996 | 0 | 16 | 0.00 |  | ENSMUSG00000023885 | 0 | 10 | 0.00 |
| ENSMUSG00000015957 | 80 | 277 | 0.00 |  | ENSMUSG00000049001 | 0 | 10 | 0.00 |
| ENSMUSG00000027276 | 4 | 32 | 0.00 |  | ENSMUSG00000028868 | 338 | 835 | 0.00 |
| ENSMUSG00000069763 | 16 | 73 | 0.00 |  | ENSMUSG00000030123 | 43 | 121 | 0.01 |
| ENSMUSG00000001761 | 26 | 101 | 0.00 |  | ENSMUSG00000016933 | 51 | 140 | 0.01 |
| ENSMUSG00000057789 | 9 | 46 | 0.00 |  | ENSMUSG00000022528 | 153 | 382 | 0.01 |
| ENSMUSG00000030605 | 213 | 650 | 0.00 |  | ENSMUSG00000031902 | 227 | 558 | 0.01 |

| **Gene ID** | **PDR_ count** | **SKYD_ count** | ***p* value** |  | **Gene ID** | **PDR_ count** | **SKYD_ count** | ***p* value** |
| --- | --- | --- | --- | --- | --- | --- | --- | --- |
| ENSMUSG00000022425 | 47 | 129 | 0.01 |  | ENSMUSG00000014932 | 0 | 18 | 0.00 |
| ENSMUSG00000024290 | 275 | 672 | 0.01 |  | ENSMUSG00000031451 | 85 | 308 | 0.00 |
| ENSMUSG00000027665 | 76 | 194 | 0.01 |  | ENSMUSG00000024526 | 7 | 44 | 0.00 |
| ENSMUSG00000006311 | 0 | 9 | 0.01 |  | ENSMUSG00000041120 | 175 | 585 | 0.00 |
| ENSMUSG00000016458 | 0 | 9 | 0.01 |  | ENSMUSG00000027500 | 0 | 16 | 0.00 |
| ENSMUSG00000026586 | 0 | 9 | 0.01 |  | ENSMUSG00000029096 | 56 | 200 | 0.00 |
| ENSMUSG00000035458 | 0 | 9 | 0.01 |  | ENSMUSG00000015619 | 42 | 156 | 0.00 |
| ENSMUSG00000031963 | 0 | 9 | 0.01 |  | ENSMUSG00000025351 | 1157 | 3589 | 0.00 |
| ENSMUSG00000057722 | 0 | 9 | 0.01 |  | ENSMUSG00000009406 | 35 | 131 | 0.00 |
| ENSMUSG00000027985 | 279 | 656 | 0.01 |  | ENSMUSG00000045817 | 241 | 759 | 0.00 |
| ENSMUSG00000004328 | 4 | 21 | 0.01 |  | ENSMUSG00000022817 | 296 | 921 | 0.00 |
| ENSMUSG00000028763 | 4 | 21 | 0.01 |  | ENSMUSG00000021253 | 5 | 34 | 0.00 |
| ENSMUSG00000020458 | 156 | 372 | 0.01 |  | ENSMUSG00000021943 | 0 | 14 | 0.00 |
| ENSMUSG00000026043 | 122 | 291 | 0.01 |  | ENSMUSG00000026842 | 27 | 101 | 0.00 |
| ENSMUSG00000026193 | 2529 | 9983 | 0.00 |  | ENSMUSG00000022521 | 80 | 252 | 0.00 |
| ENSMUSG00000000094 | 0 | 9 | 0.01 |  | ENSMUSG00000029050 | 46 | 153 | 0.00 |
| ENSMUSG00000023078 | 153 | 2061 | 0.00 |  | ENSMUSG00000020919 | 495 | 1411 | 0.00 |
| ENSMUSG00000049907 | 0 | 103 | 0.00 |  | ENSMUSG00000038400 | 78 | 240 | 0.00 |
| ENSMUSG00000021319 | 0 | 71 | 0.00 |  | ENSMUSG00000038872 | 7 | 36 | 0.00 |
| ENSMUSG00000003665 | 0 | 68 | 0.00 |  | ENSMUSG00000055653 | 39 | 125 | 0.00 |
| ENSMUSG00000036699 | 0 | 62 | 0.00 |  | ENSMUSG00000044674 | 16 | 63 | 0.00 |
| ENSMUSG00000033295 | 12 | 168 | 0.00 |  | ENSMUSG00000006958 | 0 | 12 | 0.00 |
| ENSMUSG00000024544 | 73 | 516 | 0.00 |  | ENSMUSG00000020176 | 0 | 12 | 0.00 |
| ENSMUSG00000029661 | 21 | 187 | 0.00 |  | ENSMUSG00000011096 | 189 | 520 | 0.00 |
| ENSMUSG00000025854 | 317 | 1960 | 0.00 |  | ENSMUSG00000024940 | 16 | 57 | 0.00 |
| ENSMUSG00000030669 | 0 | 36 | 0.00 |  | ENSMUSG00000024975 | 436 | 1087 | 0.00 |
| ENSMUSG00000020108 | 18 | 133 | 0.00 |  | ENSMUSG00000050071 | 0 | 10 | 0.00 |
| ENSMUSG00000021388 | 16 | 120 | 0.00 |  | ENSMUSG00000036333 | 73 | 189 | 0.01 |
| ENSMUSG00000008999 | 0 | 29 | 0.00 |  | ENSMUSG00000060216 | 563 | 1334 | 0.01 |
| ENSMUSG00000006205 | 20 | 130 | 0.00 |  | ENSMUSG00000062312 | 0 | 9 | 0.01 |
| ENSMUSG00000038508 | 20 | 127 | 0.00 |  | ENSMUSG00000026383 | 0 | 9 | 0.01 |
| ENSMUSG00000040488 | 6 | 59 | 0.00 |  | ENSMUSG00000031681 | 241 | 555 | 0.01 |
| ENSMUSG00000024598 | 0 | 24 | 0.00 |  | ENSMUSG00000033585 | 2 | 52 | 0.00 |
| ENSMUSG00000046058 | 17 | 96 | 0.00 |  | ENSMUSG00000021214 | 113 | 659 | 0.00 |
| ENSMUSG00000022150 | 264 | 971 | 0.00 |  | ENSMUSG00000006930 | 9 | 35 | 0.01 |
| ENSMUSG00000042757 | 49 | 204 | 0.00 |  | ENSMUSG00000059146 | 2 | 981 | 0.00 |
| ENSMUSG00000031074 | 0 | 20 | 0.00 |  | ENSMUSG00000029530 | 131 | 4774 | 0.00 |
| ENSMUSG00000023047 | 0 | 20 | 0.00 |  | ENSMUSG00000029379 | 0 | 159 | 0.00 |
| ENSMUSG00000034612 | 0 | 20 | 0.00 |  | ENSMUSG00000021702 | 0 | 110 | 0.00 |
| ENSMUSG00000032726 | 0 | 19 | 0.00 |  | ENSMUSG00000031780 | 217 | 2882 | 0.00 |

| **Gene ID** | **PDR_ count** | **SKYD_ count** | ***p* value** |  | **Gene ID** | **PDR_ count** | **SKYD_ count** | ***p* value** |
| --- | --- | --- | --- | --- | --- | --- | --- | --- |
| ENSMUSG00000047898 | 0 | 87 | 0.00 |  | ENSMUSG00000047379 | 174 | 416 | 0.01 |
| ENSMUSG00000042306 | 0 | 41 | 0.00 |  | ENSMUSG00000031443 | 596 | 1380 | 0.01 |
| ENSMUSG00000000869 | 1 | 49 | 0.00 |  | ENSMUSG00000009281 | 621 | 1427 | 0.01 |
| ENSMUSG00000027962 | 512 | 2993 | 0.00 |  | ENSMUSG00000023235 | 145 | 341 | 0.01 |
| ENSMUSG00000032118 | 0 | 36 | 0.00 |  |  |  |  |  |
| ENSMUSG00000048251 | 144 | 701 | 0.00 |  |  |  |  |  |
| ENSMUSG00000055994 | 3 | 47 | 0.00 |  |  |  |  |  |
| ENSMUSG00000052957 | 3 | 44 | 0.00 |  |  |  |  |  |
| ENSMUSG00000040026 | 27911 | 115085 | 0.00 |  |  |  |  |  |
| ENSMUSG00000056427 | 0 | 25 | 0.00 |  |  |  |  |  |
| ENSMUSG00000022015 | 0 | 24 | 0.00 |  |  |  |  |  |
| ENSMUSG00000021508 | 24 | 128 | 0.00 |  |  |  |  |  |
| ENSMUSG00000042190 | 205 | 820 | 0.00 |  |  |  |  |  |
| ENSMUSG00000026235 | 0 | 22 | 0.00 |  |  |  |  |  |
| ENSMUSG00000062380 | 74 | 308 | 0.00 |  |  |  |  |  |
| ENSMUSG00000037868 | 30 | 140 | 0.00 |  |  |  |  |  |
| ENSMUSG00000025959 | 134 | 517 | 0.00 |  |  |  |  |  |
| ENSMUSG00000028126 | 56 | 230 | 0.00 |  |  |  |  |  |
| ENSMUSG00000033542 | 0 | 19 | 0.00 |  |  |  |  |  |
| ENSMUSG00000019122 | 31799 | 107706 | 0.00 |  |  |  |  |  |
| ENSMUSG00000014158 | 16 | 77 | 0.00 |  |  |  |  |  |
| ENSMUSG00000041120 | 175 | 585 | 0.00 |  |  |  |  |  |
| ENSMUSG00000029371 | 0 | 16 | 0.00 |  |  |  |  |  |
| ENSMUSG00000038668 | 32 | 122 | 0.00 |  |  |  |  |  |
| ENSMUSG00000021194 | 0 | 15 | 0.00 |  |  |  |  |  |
| ENSMUSG00000028341 | 0 | 15 | 0.00 |  |  |  |  |  |
| ENSMUSG00000029071 | 30 | 115 | 0.00 |  |  |  |  |  |
| ENSMUSG00000026640 | 0 | 14 | 0.00 |  |  |  |  |  |
| ENSMUSG00000062991 | 32 | 111 | 0.00 |  |  |  |  |  |
| ENSMUSG00000020900 | 0 | 13 | 0.00 |  |  |  |  |  |
| ENSMUSG00000072596 | 4981 | 13817 | 0.00 |  |  |  |  |  |
| ENSMUSG00000037613 | 14 | 59 | 0.00 |  |  |  |  |  |
| ENSMUSG00000059714 | 877 | 2413 | 0.00 |  |  |  |  |  |
| ENSMUSG00000055633 | 6 | 31 | 0.00 |  |  |  |  |  |
| ENSMUSG00000031385 | 0 | 11 | 0.00 |  |  |  |  |  |
| ENSMUSG00000042265 | 799 | 2015 | 0.00 |  |  |  |  |  |
| ENSMUSG00000061731 | 134 | 349 | 0.00 |  |  |  |  |  |
| ENSMUSG00000019194 | 58 | 160 | 0.00 |  |  |  |  |  |
| ENSMUSG00000074657 | 0 | 10 | 0.00 |  |  |  |  |  |
| ENSMUSG00000039115 | 37 | 100 | 0.01 |  |  |  |  |  |
