# Supplementary material for: Study on the Effect of Macrophages on Vascular Endothelium in Mice With Different TCM Syndromes of Dyslipidemia and its Biological Basis Based on RNA-Seq Technology
Source: Front Pharmacol. 2021 Aug 26;12:665635. doi: 10.3389/fphar.2021.665635 (PMC8427158; doi:10.3389/fphar.2021.665635)
Supplement: Supplementary file 2 [file DataSheet1.docx]

**Supplementary File 1**

**Supplementary documentation on screening of macrophage mixed samples**

(1) Sampling and macrophage screening methods: aorta of animals from the PDR group and the SKYD group were isolated and removed intact, digested by adding trypsin, sieved and ground, digestion was terminated, centrifuged, supernatant was decanted, resuspended by adding PBS, centrifuged again, supernatant was decanted, and labeled with FITC Anti-Mouse F4/80 Antigen (BM8.1) and PE Anti-Human/ Mouse CD11b (M1/70), blown and resuspended, and stored away from light. After labeling macrophages, macrophages were screened by fluorescence-activated cell sorting (FACS) using the MoFlo XDP Ultra-Fast Flow Cell Sorting System (Beckman Coulter Co., Ltd).

(2) Pictures of screening by flow cytometry (R6 is macrophage)


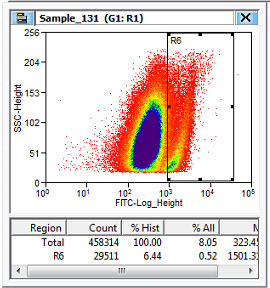

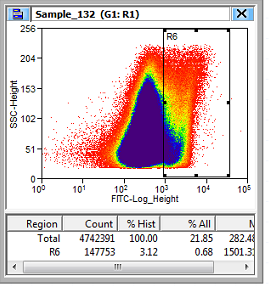


(3) Due to the insufficient number of macrophages screened from a single animal, in order to be able to carry out the next transcriptome detection, we mixed the macrophages of 5 animals in each group, which can meet the requirements of transcriptome detection;

(4) In order to ensure the objectivity and stability of statistical analysis of test data, we used the following methods for data analysis after sequencing of macrophage transcriptome:

For samples without biological duplication, the difference analysis software is edgeR software, which can realize the difference analysis of characteristic samples.

The following is the official website information of edgeR software:

<http://bioconductor.org/packages/release/bioc/html/edgeR.html>
